# Functional, transcriptomic, and lipidomic studies of the *choC* gene encoding a phospholipid methyltransferase in *Aspergillus fumigatus*

**DOI:** 10.1128/spectrum.02168-23

**Published:** 2023-11-27

**Authors:** Jiao Pan, Xinyu Yang, Cuiting Hu, Tongtong Fu, Xiuyan Zhang, Zijun Liu, Yu Wang, Fengyu Zhang, Xiaoyuan He, Jae-Hyuk Yu

**Affiliations:** 1 Institute for Cultural Heritage and History of Science and Technology, University of Science and Technology Beijing, Beijing, China; 2 Department of Microbiology, College of Life Sciences, Nankai University, Tianjin, China; 3 College of Food Science and Technology, Huazhong Agricultural University, Wuhan, China; 4 Department of Hematology, Tianjin First Central Hospital, Tianjin, China; 5 Department of Bacteriology, Food Research Institute, University of Wisconsin, Madison, Wisconsin, USA; Universidade de Sao Paulo, Sao Paulo, Brazil

**Keywords:** phosphatidylcholines, *Aspergillus fumigatus*, ChoC, transcriptomics, lipidomics

## Abstract

Phosphatidylcholines (PCs) are a class of cell membrane phospholipids playing important roles in cellular integrity and signaling. In eukaryotes, PCs are biosynthesized via the Kennedy cytidine nucleotide and the Bremer-Greenberg pathways. The *Aspergillus choC* gene is predicted to encode a phospholipid methyltransferase, the key enzyme in the latter pathway. In this study, we have characterized *choC* in the opportunistic human pathogenic fungus *Aspergillus fumigatus* and show that ChoC plays a vital role in the endogenous biosynthesis of PC, which is necessary for proper vegetative growth, development, cell viability, and cell wall integrity of the fungus. Transcriptomic analyses have revealed that the deletion (Δ) of *choC* leads to the differential expression of diverse genes related to PC biosynthetic pathways, G protein signaling pathways, cell cycle, and autophagy. Importantly, the Δ*choC* mutant averted cell death and regained cellular proliferation by regulating the expression of different PC synthesis genes after 4 days of incubation. Lipidomic analyses have demonstrated that Δ*choC* directly leads to decreased PC synthesis, thereby affecting the length and unsaturation of PC fatty acid chains. In addition, Δ*choC* leads to disturbed metabolism of other lipids, such as reduced phosphatidylethanolamine and neutral lipid triacylglycerol, but increased diacylglycerol amounts. Murine infection studies showed that invasive pathogenicity of the Δ*choC* spores was significantly weakened coupled with a drastic reduction of their ability to colonize in kidneys. In summary, ChoC plays central roles in PC biosynthesis, lipid metabolism, cell membrane/wall integrity, endoplasmic reticulum function, and pathogenicity in *A. fumigatus*, implying that ChoC can be a novel target for antifungal therapy.

**IMPORTANCE:**

This study explored the phospholipid metabolic pathway in *A. fumigatus* and its relationship with fungal growth, metabolism, and pathogenicity. ChoC, based on its critical roles in many aspects of the fungus and relatively conserved characteristics in filamentous fungi with low similarity with mammalian ones, can be a novel target of new antifungal drugs.

## INTRODUCTION

The filamentous ascomycete *Aspergillus fumigatus* is an opportunistic human pathogenic fungus that causes severe and fatal invasive infections in immunocompromised individuals (1). *A. fumigatus* produces specialized asexual reproductive structures called conidiophores, which bear a large number of asexual spores called conidia (2). Conidia are highly thermally tolerant and can survive even at temperatures above 50°C, which confers the fungus’ enhanced fitness in decaying vegetation, a frequently inhabited niche (3, 4). The small size of *A. fumigatus* conidia (2–3 µm) is ideal for infiltrating deep into the respiratory tract, which is usually the primary route of infection. The conidial surface comprises hydrophobin and melanin, which block the exposure of the pattern recognition receptor β−1,3-glucan and α-mannose on the spore wall (5 – 13). During germination, the swelling conidia and expanded hyphae are recognized by host immune cells, which in turn secrete defensive enzymes, such as superoxide dismutase and catalase, and eliminate the reactive oxygen species (ROS) produced by immune cells (14, 15). *A. fumigatus* produces the mycotoxins gliotoxin (16 – 19) and ergot alkaloids, which inhibit immune effectors and induce cell apoptosis. These virulence factors are key weapons for *A. fumigatus* to infect host cells (20 – 23).

Numerous lipid molecules exist in nature, with diverse chemical structures and physical properties. In 2005, to encourage development in the field of lipidomics, the International Lipid Classification and Nomenclature Committee divided lipids into eight categories: fatty acids (FA), glycerolipids (GL), glycerophospholipids (GP), sphingolipids (SL), sterol lipids (ST), saccharolipids, prenol lipids (PR), and polyketides (PK). FA, GP, SL, and ST are common lipid molecules in most cells, whereas saccharolipids, PR, and PK are found mainly in bacteria, fungi, and plants (14). Approximately, 5% of the genes in eukaryotic cells is related to the metabolism of lipid molecules (24) owing to the wide variety of lipid molecules and their essential roles in cells. Thus, it is anticipated that targeted interference of fungal lipid metabolism can help fight against pathogenic fungi. As a base, understanding the specific lipid metabolism is important.

Phosphatidylcholines (PCs) are a group of major phospholipid molecules in the biomembrane, accounting for approximately 50% of membrane phospholipids in most eukaryotic cells (25, 26). Phosphorylcholine occupies the sn-3 position of the glycerol backbone, and two fatty acyl chains are linked to the sn-1 and sn-2 positions, the length and degree of saturation of which determine the species of PC (27). The length of the fatty acyl chains of most PCs is usually around 14–26, with 16 or 18 carbon atoms being the most common (28). In yeast cells, a steady drop in PC content leads to shortened PC chain length and a lower degree of unsaturation to maintain membrane fluidity and bilayer properties (29). The number of double bonds in the fatty acyl chain determines the shape of the PC and, therefore, affects the physical properties of the biomembrane.

In mammalian cells (except the liver cells), the CDP-choline pathway is the only *de novo* biosynthetic pathway for PC (30, 31). However, in *Saccharomyces cerevisiae*, PC is mainly synthesized by the CDP-DAG and CDP-choline pathways, both of which are responsible for maintaining balanced levels of PC in cells (32 – 34). The CDP-DAG pathway utilizes the precursor phosphatidic acid, produced by phospholipids, following three subsequent methylation reactions. Exotic choline is phosphorylated by choline kinase, which is the first step in PC generation via the CDP-choline pathway (35, 36). However, studies about PC biosynthesis in filamentous fungi are limited. In *Pyricularia oryzae*, *Botrytis fabae*, and *Fusarium graminearum*, the blockage of PC synthesis indirectly inhibits chitin generation (37). In *Aspergillus nidulans,* PC content has a critical impact on hyphal elongation and morphology (38). ChoB, a phosphatidylserine (PS) decarboxylase, catalyzes the conversion of PS to phosphatidylethanolamine (PE) (24). Both *choA* and *choC* are predicted to encode methylases that function in the successive addition of methyl groups to PE, thereby producing PC (39). The deletion of *choC* in *A. nidulans* limits vegetative growth, significantly reduces cell viability, causes severe defects in asexual development, and induces pre-apoptosis (38, 40). In this study, to understand the role of the *choC* gene in *A. fumigatus*, we carried out functional, transcriptomic, and lipidomic studies and further explored the synthesis of PC and its metabolic regulatory mechanisms.

## RESULTS

### Functional characterization of the *choC* gene in *A. fumigatus*

**Fig 1:**
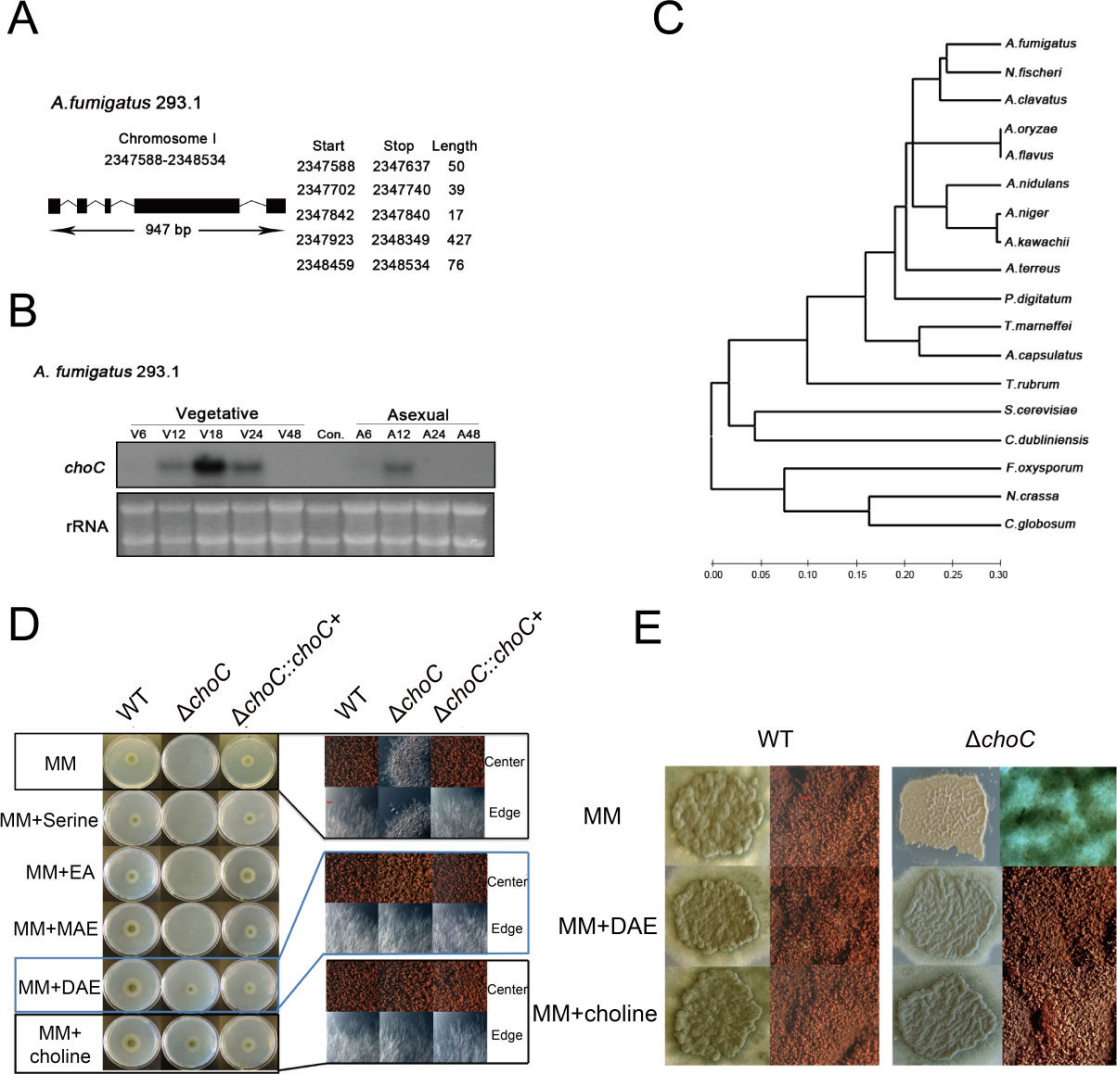
Summary and function of *choC.* (**A**) Identification of the gene *choC*. (**B**) Levels of *choC* mRNA during vegetative growth and asexual development. (**C**) ChoC is conserved among most fungi. (**D**) Phenotypes of the colony of wild-type (WT), Δ*choC*, and complemented strains on solid minimal medium (MM), MM + serine, MM + ethanolamine (EA), MM + monoethanolamine (MAE), MM + diethanolamine (DAE), and MM + choline upon incubation at 37°C for 3 days. Scale bar: 10 µm. (**E**) Hyphae of WT, Δ*choC*, and complemented strains grown in solid MM. Scale bar = 10 µm.

The structure of the *choC* gene was determined by reverse transcription polymerase chain reaction, and the genomic DNA and cDNA sequences were compared. The *choC* coding region comprises 947 bp, composed of five exons and four introns (Fig. 1A), and the predicted ChoC protein is 203 amino acids long. Northern blot analysis indicated that high levels of *choC* mRNA accumulated after 18 h of vegetative growth and 12 h of asexual development (Fig. 1B). The predicted ChoC protein is highly conserved in most fungi (Fig. 1C).

To verify the function of *choC*, we generated the Δ*choC* mutant through the double-joint polymerase chain reaction (DJ-PCR) method using *A. nidulans pyrG* as the marker. We also generated complemented strains of *A. fumigatus* via pPTR1 plasmid, re-introducing the *choC* wild-type (WT) allele into the deletion mutant separately. As shown in Fig. 1D, the Δ*choC* mutant exhibited severely impaired vegetative growth and lacked asexual development on day 3 in minimal medium (MM). The addition of diethanolamine (DAE) or choline, but not serine, ethanolamine (EA), or monoethanolamine (MAE), completely restored vegetative growth of the Δ*choC* mutant (Fig. 1D). However, when DAE or choline was added, the Δ*choC* mutant displayed near-complete restoration of conidiation (Fig. 1E). These results indicate that the functions of *choC* are conserved in *A. fumigatus* and are involved in the last two steps of the Bremer-Greenberg methylation pathway, from phosphoryl monoethanolamine to phosphoryl diethanolamine (PDAE) and PDAE to PC. PC is essential for vegetative growth and development of *Aspergillus* species.

### ChoC is necessary for proper proliferation, cell viability, and cell wall integrity in *A. fumigatus*

**Fig 2:**
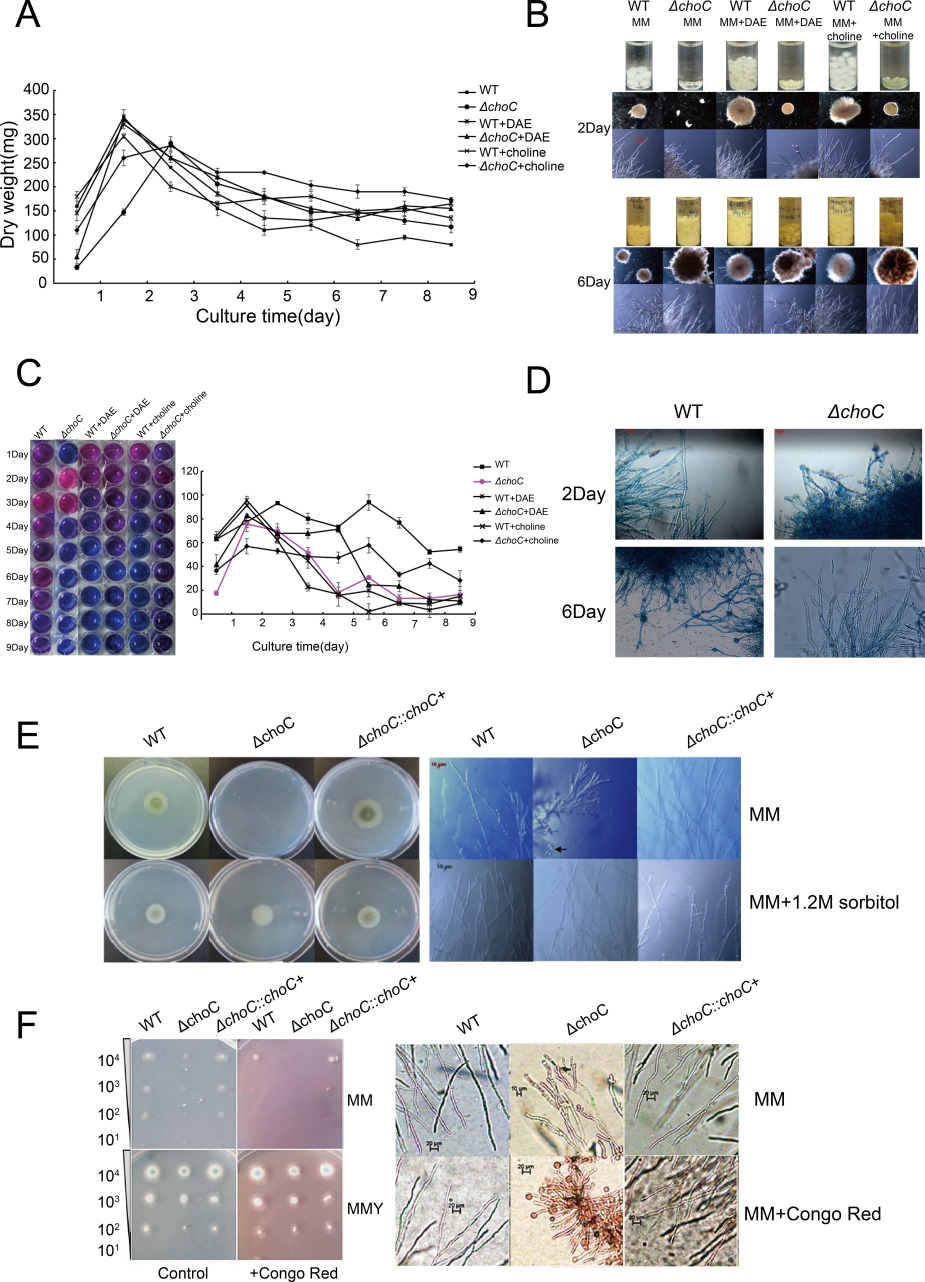
Roles of choC in cell viability, cell death, and cell wall integrity. (**A**) Mycelial dry weights of WT and Δ*choC* strains grown in liquid MM, MM + DAE, and MM + choline at 37°C for 9 days. Data are the mean values of three independent experiments. (**B**) Phenotypes of mycelial aggregates formed in liquid MM at days 2 and 6. Scale bar: 20 µm (middle panels) and 10 µm (bottom panels). (**C**) Alamar blue reduction rates of WT and Δ*choC* strains grown in liquid MM, MM + DAE, and MM + choline at 37°C for 9 days. Data points are the mean of three independent experiments. (**D**) Apoptosis analyses of WT and Δ*choC* strains grown in liquid MM by Evans blue staining. Scale bar is 5 µm. The photographs were taken on days 2 and 6. (**E**) Sensitivity to osmotic pressure of WT and Δ*choC* strains grown in liquid MM with 1.2 M sorbitol. Scale bar is 10 µm. (**F**) Effects of Congo Red on the growth of the Δ*choC* mutant. Scale bar: 10 and 20 µm.

To further determine the role of *choC* in cell proliferation, we examined the mycelial dry weight of the WT (*A. fumigatus* 293.1) and Δ*choC* strains in liquid MM, MM + DAE, and MM + choline. As shown in Fig. 2A, the mycelial dry weight of the WT strain reached a maximum on day 2 in MM, while the dry weight of Δ*choC* strains reached a maximum on day 3 and showed severely restricted vegetative growth during the first 2 days of incubation. After the addition of DEA and choline, the vegetative growth of Δ*choC* was almost restored. Moreover, the incubation of the Δ*choC* mutants in liquid MM led to the formation of large mycelial aggregates with protruding hyphae on day 6 (Fig. 2B). To evaluate the role of *choC* in cell viability (measured by mitochondrial activity), we determined the reduction rates of alamar blue from WT and Δ*choC* strains grown in liquid MM, MM + DAE, and MM + choline medium. WT strains reached a peak in cell viability on day 3 and then a slow decline in cell viability was observed from day 3 to day 6 in MM, while the Δ*choC* mutant cells displayed high cell viability on day 2, but a sharp reduction in cell viability from day 3 to day 6. After adding DAE and choline, the Δ*choC* mutant cells regained viability, which was comparable to that of WT (Fig. 2C). We also determined cell death (apoptosis) levels in WT and Δ*choC* strains using Evans blue staining. As shown in Fig. 2D, compared to WT, Δ*choC* strains displayed precocious cell death on day 2 in MM, while on day 6 Δ*choC* strains could escape cell death and regenerate new viable hyphae from big mycelial aggregates. To test whether the absence of *choC* affected the integrity of the cell wall, we investigated the hyphal morphology of the Δ*choC* mutant cells in MM containing 1.2 M sorbitol and found that the swollen hyphae of the Δ*choC* mutant were partly restored by osmotic stabilizers and normal hyphae were generated (Fig. 2E). When 250 mg/L Congo red was added to MM, the Δ*choC* mutant grew less well than WT and more swollen structures appeared at the tips of the mutant hyphae (Fig. 2F), suggesting that hyphal tip swelling was likely caused by defective cell walls. These results indicate that *choC* deficiency affects cell proliferation, viability, death, and wall integrity in *A. fumigatus* and that the function of *choC* is conserved in *A. fumigatus*.

### Genome-wide expression analyses of the Δ*choC* mutant

The *choC* gene encodes an important methylation enzyme essential for *A. fumigatus* to synthesize PC. To explore the genes and gene networks involved in cell proliferation, cell death, and cell reprogramming with the disturbance of PC homeostasis, we performed RNA-Seq analyses of WT and the Δ*choC* mutant on days 2 and 4 of cultivation in MM (Table S1).

**Fig 3:**
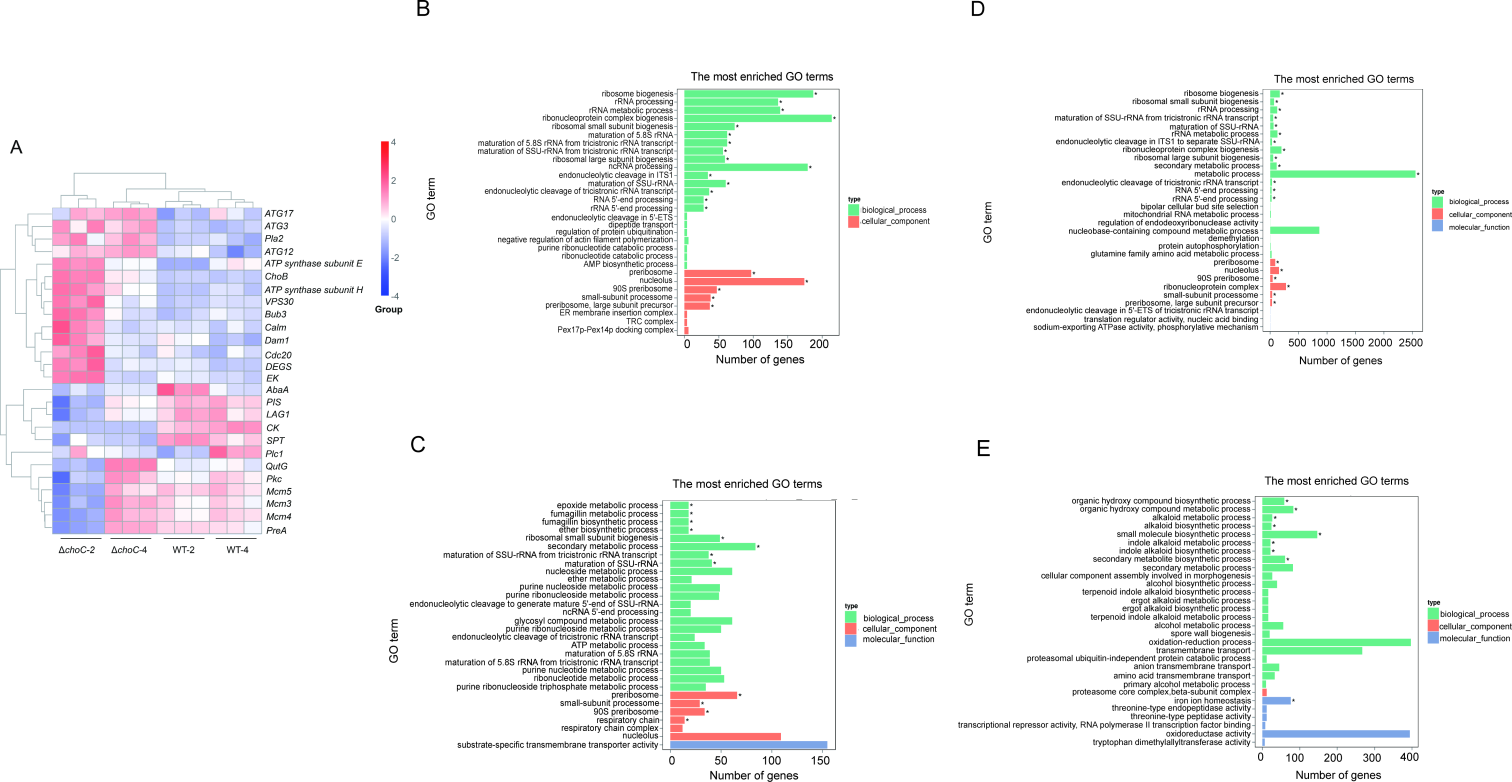
DEGs and gene ontology (GO) enrichment analyses. (**A**) Heatmap analysis shows that statistical significance in some important genes in phospholipid metabolism, cell cycle, autophagy, and other metabolic pathways in WT-2,Δ*choC*-2 and WT-4,Δ*choC*-4 strains. WT-2/Δ*choC*-2 means WT/Δ*choC* strains were cultured in MM for 2 days. WT-4/Δ*choC*-4 means WT/Δ*choC* strains were cultured in MM for 4 days. Triplicates were analyzed per strain per time point. (**B**) GO enrichment analysis of DEGs between Δ*choC*-2 and WT-2. (**C**) GO enrichment analysis of DEGs between Δ*choC*-4 and WT-4. (**D**) GO enrichment analysis of DEGs between Δ*choC*-4 and Δ*choC*-2. (**E**) GO enrichment analysis of DEGs between WT-4 and WT-2. The ordinate is an enriched GO term, and the abscissa is the number of DEGs in the term. Different colors are used to distinguish three major categories: biological processes, cell components, and molecular functions. "*" is a significant enrichment of GO term.

We found 4,903 differentially expressed genes (DEGs), of which 2,360 were significantly upregulated, whereas 2,543 were significantly downregulated in the Δ*choC* mutant on day 2 (Fig. S1A). Whereas on day 4, of 3,486 DEGs, 1,710 were significantly upregulated and 1,776 were significantly downregulated in the Δ*choC* mutant (Fig. S1B). Comparing the Δ*choC* mutant cells of 4 and 2 days of cultivation in MM, of 5,060 DEGs, 2,520 were significantly upregulated and 2,540 genes were significantly downregulated (Fig. S1C). Comparing the WT cells of days 4 and 2, from 3,464 DEGs, 1,629 were significantly upregulated and 1,835 were significantly downregulated (Fig. S1D). Significant differences were found among these DEG-encoded proteins involved in metabolic pathways such as glycerophospholipid metabolism (*CK*, *ChoB*, *EK*, and *Pla2*), phosphatidylinositol signaling system (*PIS*, *Pkc*, *Plc1*, *Calm*, and *QutG*), MAPK signaling system (*AbaA* and *PreA*), cell cycle (*Mcm5*, *Mcm3*, *Mcm4*, *Bub3*, *Cdc20*, and *Dam1*), sphingolipid metabolism (*SPT* and *DEGS*), autophagy (*ATG17*, *VPS30*, *ATG3*, and *ATG12*), and oxidative phosphorylation (vacuolar ATP synthase subunit H and ATP synthase subunit E) indicating that the lack of *choC* function disrupted PC synthesis and has a high impact on the synthesis of lipids, proteins, and signal regulation of metabolic pathways (Fig. 3A; Fig. S2; Table S3).

To further understand the functions of these DEGs, gene ontology (GO) term enrichment analysis (*P* < 0.05) was performed using GOseq. Based on the sequence homology, the identified genes were grouped into categories based on three common biological properties: biological processes, cellular components, and molecular function (41). The Δ*choC-2* vs WT-2 analysis showed the enrichment of preribosome (*P* = 2.62 × 10^−21^), nucleolus (*P* = 2.48 × 10^−13^) in the cellular component, ribosome biogenesis (*P* = 3.91 × 10^−18^), rRNA processing (*P* = 2.61 × 10^−12^), and ncRNA processing (*P* = 4.66 × 10^−8^) in the biological process. Most of the related genes in these two categories were downregulated in the Δ*choC*-2 cells (Fig. 3B). The Δ*choC*-4 vs WT-4 analysis showed enrichment of preribosomes (*P* = 1.17 × 10^−5^), small-subunit processomes (*P* = 0.001) in the cellular component, and epoxide metabolic processes (*P* = 0.001), fumagillin metabolic processes (*P* = 0.001), and fumagillin biosynthetic processes (*P* = 0.001) in the biological process. Most of the related genes in these two categories were downregulated in the Δ*choC-*4 cells (Fig. 3C). The Δ*choC*-4 vs Δ*choC*-2 analysis showed enrichment of preribosome (*P* = 3.16 × 10^−10^), nucleolus (*P* = 6.46 × 10^−5^) in the cellular component and ribosome biogenesis (*P* = 4.82 × 10^−7^), rRNA processing (*P* = 4.04 × 10^−5^), maturation of SSU-rRNA (*P* = 6.46 × 10^−5^) in the biological process. Most of the related genes in these two categories were upregulated in the Δ*choC-*4 cells (Fig. 3D). The analysis of WT-4 vs WT-2 showed enrichment of the organic hydroxy compound biosynthetic process (*P* = 0.009), alkaloid metabolic process (*P* = 0.009), indole alkaloid metabolic process (*P* = 0.02), secondary metabolite biosynthetic process (*P* = 0.02), and iron ion binding (*P* = 0.02) in molecular function. Most related genes in these two categories were downregulated in WT-4 cells (Fig. 3E).

### Disruption of PC synthesis has a negative impact on energy metabolism, material transformation, and signal regulation in Δ*choC*

**Fig 4:**
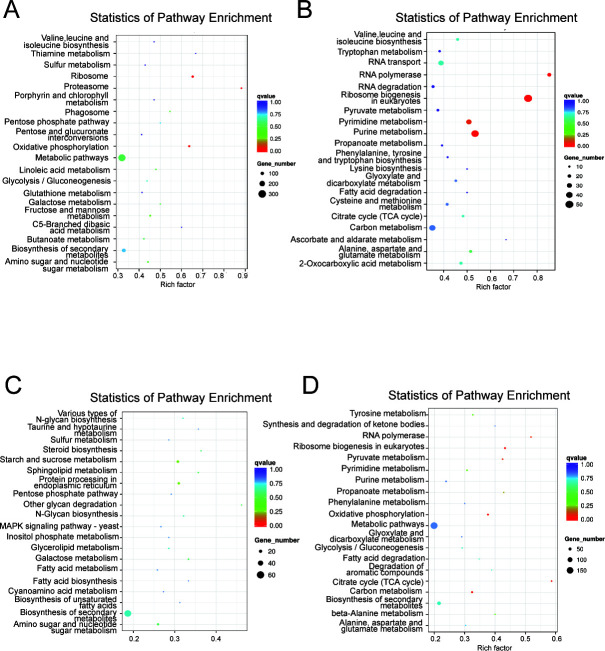
Statistical analysis of KEGG pathway enrichment. (**A**) The metabolic pathways of significantly upregulated DEGs in the Δ*choC* mutant at 2 days. (**B**) The metabolic pathways of significantly downregulated DEGs in the Δ*choC* mutant at 2 days. (**C**) The metabolic pathways of significantly upregulated DEGs in the Δ*choC* mutant at 4 days. (**D**) The metabolic pathways of significantly downregulated DEGs in the Δ*choC* mutant at 4 days. Rich factor refers to the ratio of the number of differentially expressed genes enriched in the pathway to the number of annotated genes. The greater the “Rich factor,” the greater is the degree of enrichment; *q*-value is the *P*-value after multiple hypothesis test corrections. The *q*-value ranges within (0, 1), and the closer to 0, the more significant the enrichment is. The first 20 entries that show the most significant enrichment are shown.

To determine the DEGs involved in the major biochemical, metabolic, and signal transduction pathways, KOBAS software was used to test the statistical enrichment of DEGs in the Kyoto Encyclopedia of Genes and Genomes (KEGG) pathways (Fig. 4A through D) (42, 43). Genes with significant differences in the expression of KEGG pathways among the four comparative combinations are shown in Tables S5 to S8 (log_2_FC > 0.5, *P* < 0.05). On day 2, the significantly upregulated DEGs in the Δ*choC* cells were mainly involved in primary metabolic pathways related to energy metabolism, such as oxidative phosphorylation and the pentose phosphate pathway; the biosynthesis pathway of amino acids such as valine, linoleic acid, and butyric acid; and other pathways related to lipid metabolism and the synthesis of secondary metabolites. The downregulated genes mainly focused on the biosynthesis of amino acids, the assembly of ribosomes, and other pathways related to protein metabolism, the metabolism of purine, pyrimidine, and RNA, and the biosynthesis of fatty acids. On day 4, the significantly upregulated DEGs were mainly involved in lipid metabolism, such as the biosynthesis and metabolism of unsaturated fatty acids and other fatty acids; the metabolic pathways of sphingolipids, steroids, and glycerides; the occurrence of proteins in the endoplasmic reticulum (ER); the metabolic pathways related to glucan synthesis; and the signal metabolic pathways, such as inositol phosphate and MAPK signaling pathways. It was noticeable that *CK1*, encoding choline kinase, in the Δ*choC* mutant practically had no mRNA levels compared to WT on days 2 and 4, as shown in Tables S5 and S6. According to the results of the KEGG experiment, we hypothesized that *CK* genes may be closely related to *choC* and knocked out two genes, *CK1* and *CK2,* in WT and the Δ*choC* mutant, respectively. Growth of Δ*CK1* and Δ*CK2* strains was slightly reduced compared to that of WT strains, indicating that the deletion of *CK1* or *CK2* in WT has a minor impact on *A. fumigatus*. However, growth Δ*CK1* and Δ*CK2* was significantly better than that of the Δ*choC* mutant on solid MM. The Δ*choC*Δ*CK1* double mutant grew poorly on MM even with the choline supplementation or on minimal medium with yeast extract (MMY). This result indicates that the absence of CK1 and ChoC functions has a synergistic adverse effect on *A. fumigatus* growth (Fig. S3).

### Lipidomic analyses of *choC* function in *A. fumigatus*

**Fig 5:**
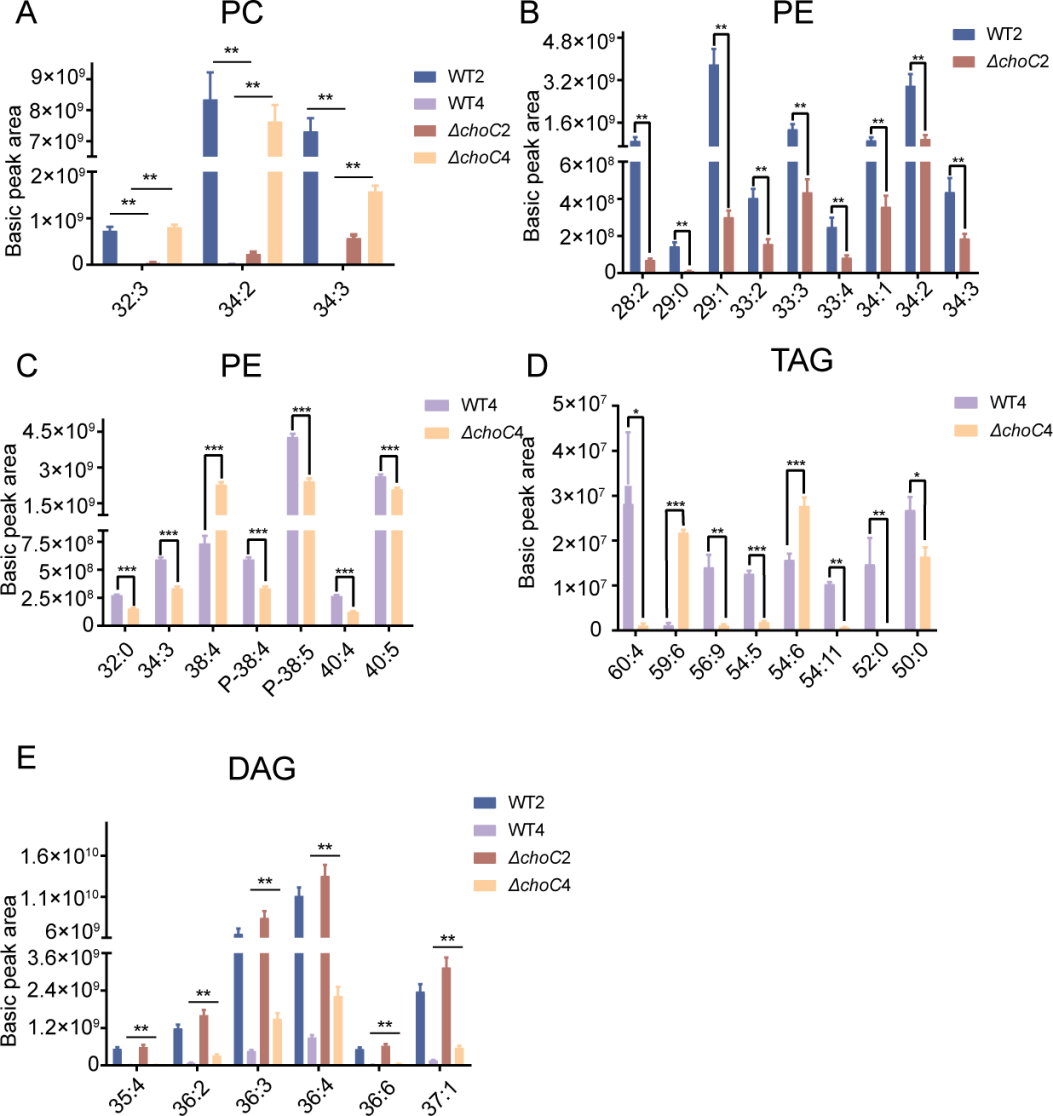
Influence of *choC* on lipid metabolism in *A. fumigatus.* (**A**) The abundance of PC in WT and Δ*choC* strains at days 2 and 4. (**B**) The abundance of PE in WT and Δ*choC* strains at day 2. *N* = 6, *P < 0.05; **P < 0.01; and ***P < 0.001. (**C**) The abundance of PE in WT and Δ*choC* strains at day 4. *N* = 6, *P < 0.05; **P < 0.01; and ***P < 0.001. (**D**) The abundance of TAG in WT and Δ*choC* strains at day 4. (**E**) The abundance of DAG in WT and Δ*choC* strains at days 2 and 4.

The synthesis and catabolism of different types of lipids cross each other, particularly in lipid synthesis. PE is an intermediate metabolite of the methylation pathway that synthesizes PC and is closely related to the two main pathways of PC synthesis. Knockout of *choC* not only affects the synthesis of PC but also interferes with the balance of PE metabolism. To assess the effect of Δ*choC* on the lipid profile of *A. fumigatus*, we systematically analyzed the lipidomes of Δ*choC* strains compared to that of WT strains cultured for 2 and 4 days in MM, respectively. Detailed information for each group is listed in Table S2. We harvested the mycelium of WT strains and Δ*choC* strains cultivated in liquid MM on days 2 and 4. The results showed that the PC content in the WT strain was significantly reduced on day 4 compared to that on day 2, and most of the reduced PCs produced fatty acyl chains with 32 and 34 carbons (Fig. S4A). The Δ*choC* mutant showed the opposite results. The PC contents of the mutant were much higher after culturing for 4 days than after 2 days (Fig. S4B). The PC content of WT strain was higher than that of the Δ*choC* mutant (Fig. 5A). On day 2, the content of PEs (28:2, 29:0, 29:1, 33:2, 33:3, 33:4, 34:1, 34:2, and 34:3) in the Δ*choC* mutant was lower than that in WT strain (Fig. 5B). On day 4, the PE content in the mutant generally decreased compared to that in WT, except for the PE molecule (38:4) (Fig. 5C). These results indicate that the loss of *choC* disturbs PE anabolism and catabolism. Although the expression of many genes in the PE metabolic pathway was upregulated, the content of PEs in the Δ*choC* mutant was significantly reduced. The abundance of intracellular neutral lipids was also significantly affected in the Δ*choC* mutant. The TAG content in the Δ*choC* mutant was significantly lower than that in WT on day 4, except for the TAG molecules (59:6 and 54:6) (Fig. 5D). The abundance of DAG, which comes from the breakdown of TAG, in the Δ*choC* mutant remarkably increased compared to that in WT at both time points (Fig. 5E). Combined with changes in PCs and PEs, Δ*choC* led to the inhibition of phospholipid biosynthesis. It is necessary to mobilize neutral lipids stored in cells. As the decomposition of TAG can provide fatty acyl chains for the synthesis of lipids, the TAG content in the Δ*choC* mutant decreased, and accordingly, the content of DAG, the degradation product of TAG, increased significantly.

### ChoC is required for the pathogenesis of *A. fumigatus*

**Fig 6:**
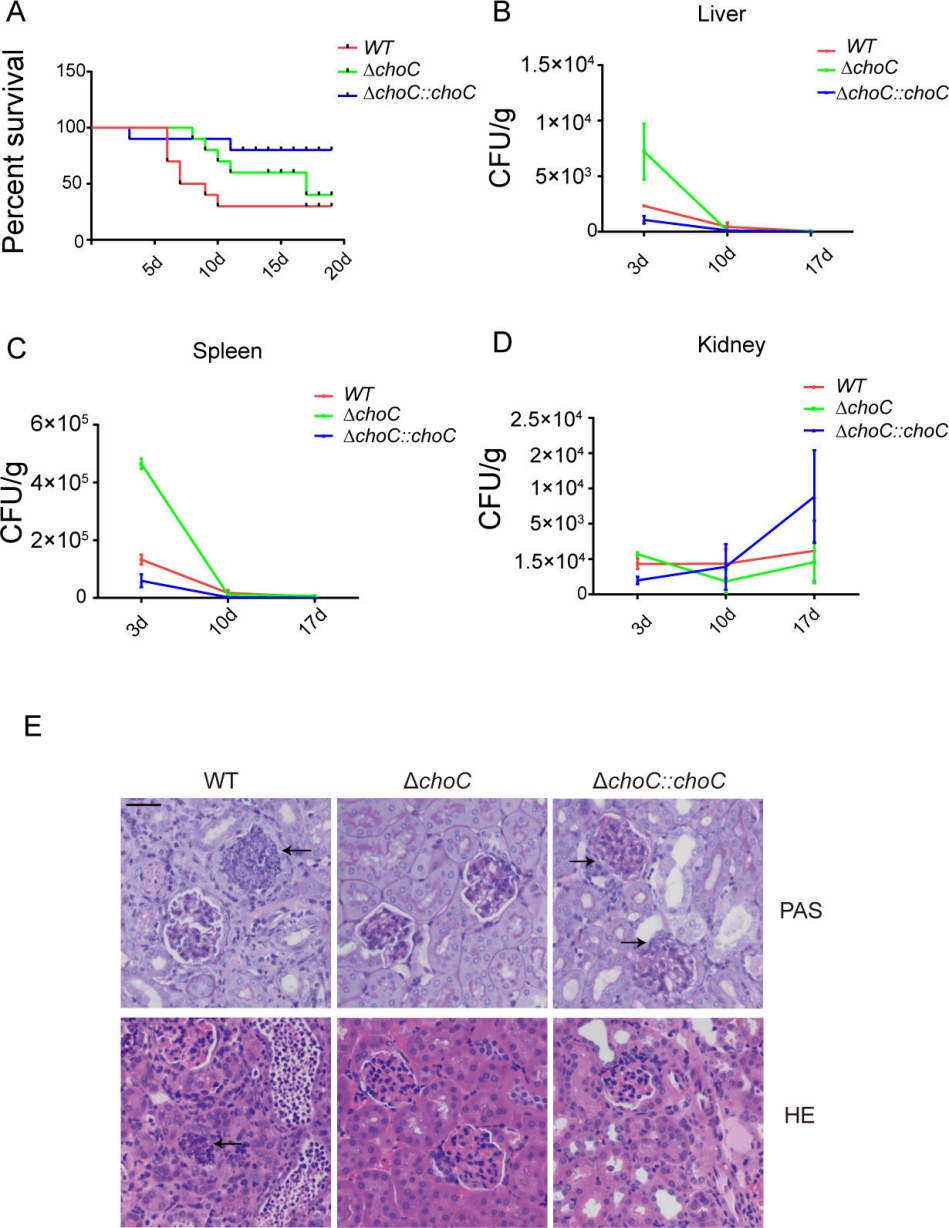
Requirement of ChoC for the pathogenesis of *A. fumigatus*. (**A**) Survival curve of mice infected with *A. fumigatus* in 3 weeks. (**B**) Fungal burden of the liver in mice infected with *A. fumigatus* at days 3, 10, and 17. (**C**) Fungal burden of the spleen in mice infected with *A. fumigatus* at days 3, 10, and 17. (**D**) Fungal burden of the kidney in mice infected with *A. fumigatus* at days 3, 10, and 17. (**E**) Representative images of periodic acid-Schiff (PAS) and hematoxylin-eosin (HE)-stained kidney sections (200×) on day 23. Bar = 100 µm for 200.

Since the viability of the Δ*choC* mutant was much lower than that of WT with early onset of apoptosis, we examined the effects of *choC* on the pathogenesis of *A. fumigatus*. Mice were infected with 3 × 10^6^ conidia of WT, the Δ*choC* mutant, and complemented strains by tail vein injection and observed daily. The survival curve of the mice showed that almost all mice survived on day 5, and most died between days 5 and 10. The virulence of mutant strain was much lower than that of WT strain, and the invasive lethality in mice was significantly reduced (Fig. 6A). Simultaneously, we examined the fungal burden in the liver (Fig. 6B), spleen (Fig. 6C), and kidneys (Fig. 6D) of mice at 3, 10, and 17 days after infection. After the spores entered the body, the immune system of the mice responded quickly, and the spores in the liver and spleen were cleared within 3–10 days. Over time, these spores gradually transferred to and colonized the kidney. The number of spores in the kidney gradually increased in mice infected with conidia of WT and complemented strains. For the mutant strains, the number of conidia entering the kidney was higher than that of the WT and complementation strains at day 3, but decreased significantly and was lower than that of the WT strains at days 10 and 17. These indicate that the deletion of *choC* reduced the ability of *A. fumigatus* conidia to colonize the kidney, making it more likely to be cleared by immune cells in the blood.

Histopathological examination of the kidneys obtained from mice infected with WT, mutant, and complemented strains was performed at three selected time points: 3, 13, and 23 days. At day 23, periodic acid-Schiff (PAS) and hematoxylin-eosin (HE) staining showed that the kidney tissues infected with WT strains showed severe damage after the invasion of spores and a swollen disrupted glomerulus (Fig. 6E). Significant inflammatory cell infiltration was observed in the kidneys of mice infected with WT and complemented strains, indicating a strong immune response caused by immune cells phagocytizing the spores. However, the Δ*choC* conidia did not induce a significant immune response in host cells. This indicates that the loss of *choC* reduces the virulence of *A. fumigatus* spores.

## DISCUSSION

Our comprehensive functional studies have revealed that *choC* plays a pivotal role in cell viability, asexual reproduction, and cell death in *A. fumigatus*. These results are consistent with our previous studies done in the model fungus *Aspergillus nidulans* (26). Furthermore, our multi-omics studies have elucidated the molecular mechanism by which ChoC controls the synthesis of PCs and regulates the growth and metabolism of *A. fumigatus*.

The MCK1-Cdc6 pathway is recognized in yeast cells to prevent DNA replication in time after damage to the plasma membrane, which ensures that DNA replication occurs only when cell wall integrity is guaranteed (44). Insufficient PC synthesis has a great influence on the vegetative growth of *A. fumigatus* because of the vital role of PC in the membrane structure. Loss of *choC* directly led to the reduction of PC biosynthesis in *A. fumigatus*, and the PC content in the Δ*choC* mutant was significantly lower than that of WT, but only in 2 days rather than 4 days according to our study. The colony diameter of the Δ*choC* mutant on solid MM was much smaller than that of WT because the extension of hyphae requires a large amount of PC. The lack of *choC* interrupted the CDP-DAG pathway for intracellular PC synthesis, and the supply of PC was greatly blocked, resulting in a weakening of the growth dynamics of the hyphae (45).

Through metabolomics analyses, when cultivated in the MM for 2 and 4 days, the deletion of *choC* completely inhibited the expression of the choline kinase CK1 in the CDP-choline pathway, indicating that in *A. fumigatus*, the two PC synthesis pathways were closely related (46). When both the *CK1* and *CK2* genes were knocked out, it did not significantly impact the growth and development of *A. fumigatus* as severe as the *choC* deletion. It is possible that choline kinase is not a rate-limiting enzyme in the CDP-choline pathway and that there are two isoenzymes, CK1 and CK2, which are responsible for the phosphorylation of choline in *A. fumigatus*. Their individual deletions did not affect fungal cells as severe as the lack of *choC*. We did not obtain double-gene deletion strains for *choC* and *CK2* after several attempts. It was suspected that the *CK2* gene might play an indispensable role in the growth of *A. fumigatus* in the absence of *choC*.

The ER is the leading site of phospholipid synthesis in cells (47). When *choC* is knocked out, inhibition of PC synthesis causes an imbalance in lipid levels. According to the transcriptome results of mutant strains cultured in MM on day 2, we found that the imbalance in PC synthesis caused by the deletion of *choC* gene induced the ER stress response, and mRNA levels of genes involved in protein synthesis, secretion, transmission between membrane vesicles, protein lysinization, and degradation in the ER changed significantly (48). Almost all proteins of ribosomal subunits, some genes related to the protein-directed transport between the endoplasmic reticulum and Golgi apparatus, and the endoplasmic reticulum and mitochondria, as well as membrane vesicles, also showed a significant upward trend (49). These data suggest that the synthesis and catabolism of proteins in cells are accelerated by Δ*choC*, which further leads to ER stress and triggers the unfolded protein response (UPR) (50). If endoplasmic reticulum stress is not properly resolved, UPR leads to cell death (51, 52).

In eukaryotes, the glycosylphosphatidylinositol (GPI) anchor is a relatively conserved structure that is an important post-translational modification of many cell surface proteins (such as enzymes, receptors, and adhesion factors), transferring these proteins from their synthesis sites to their final residence (36). Bruneau et al. identified nine GPI anchor proteins in *A. fumigatus*, of which five (Csa1p, Crh1p, Crh2p, Ecm33p, and Gas1p) had homologs in yeast cells (53). Genetic experiments have shown that these proteins play important roles in the morphogenesis of cell wall (54). The addition of Congo red blood cell sensitizer and transcriptome results confirmed that the normal synthesis of the GPI anchor would be affected when PC synthesis was disordered. The GPI anchor was closely connected to the cell wall, and its abnormality would undoubtedly affect the integrity of the cell wall. In addition, the inhibition of GPI anchor synthesis in *A. fumigatus* leads to abnormal mycelial growth and asexual development (36). It has been found that the virulence of gene-defective strains in immune-deficient mice is also reduced by co-infection with normal and gene-defective strains (55). For pathogenic fungi, the cell wall is crucial for their virulence and pathogenicity, which not only endows fungal cells with the ability to adhere to host tissues but also protects cells from the influence of the host defense system (56). Therefore, the absence of *choC* negatively impacts the growth, reproduction, and cytotoxicity of *A. fumigatus*.

We found that the lack of *choC* affected cell growth. However, there is a precise and delicate strategy for the Δ*choC* mutant to exert limited resources under the conditions of nutrient insufficiency. Lipid droplets (LDs) store excess fatty acids, such as TAG and steryl esters (STE), to prevent them from poisoning cells. These two neutral lipids can be synthesized by transferring acyl groups from fatty acids or phospholipids to DAG or sterols. In eukaryotic cells, excess fatty acids are stored in lipid droplets. Autophagy is triggered when the cells are in a state of starvation. Lysosomes and LDs have the function of converting TAG and STE into fatty acids and cholesterol to provide the nutrients needed for growth and to maintain the balance of lipids (57). Loss of the *choC* gene disrupts the CDP-DAG pathway for PC synthesis in cells, resulting in impaired synthesis of PC. In the glycerolipid metabolism pathways, mRNA levels of the gene encoding TAG lipase increased 1.0 time, which was in parallel with the lipidomic results that the TAG content in the Δ*choC* mutant was significantly reduced at day 2 compared with WT, and DAG levels increased accordingly. DAG is not only an important intermediate of the phospholipid metabolic network but also an intracellular secondary messenger molecule that is involved in the phosphatidylinositol signaling pathway, MAPK signaling pathway, etc., regulating physiological activities to cope with pressure inside or outside the cell (58 – 60). We believe that the Δ*choC* mutant uses this mechanism of lipid autophagy to maintain intracellular lipid homeostasis and survive.

In fungi, the CDP-DAG and CDP-choline pathways cooperate to ensure PC biosynthesis in fungal cells. By knocking out *choC*, we completely eliminated the CDP-DAG pathway and disrupted the homeostasis of PC metabolism, thus triggering various cellular changes. After finding abnormal conditions, the cell’s first response is to correct them immediately. The biosynthesis of PC in the Δ*choC* mutant was restored through the CDP-choline pathway with the absorption of exogenous choline, and the mutant almost completely regained the normal growth levels of WT when choline was added to MM (61). Although there is no choline supplement in MM, *A. fumigatus* can use limited resources to increase PC synthesis. According to the transcriptomic results, in the glycerophospholipid metabolism pathway (Fig. S5), mRNA levels of the genes encoding the PS decarboxylase (4.1.1.65) increased 4.8 times, while the lipidomic results showed that the content of PE in the Δ*choC* mutant after 2 days of cultivation decreased. This may be because the degradation rate was much higher than the generation rate. Transcript levels of genes encoding phospholipase A2 PlaA (3.1.1.4), which degrades PE to LPE, and lysophospholipase Plb3 (3.1.1.5), which transfers LPE to glycerol-3-phosphoethanolamine, increased and the gene (2.7.1.82) responsible for the phosphorylation of ethanolamine increased 1.8 times. All the data display another pathway (as shown in Fig. S5 with the red curve) for PC synthesis other than the two known pathways. Phosphoethanolamine is added to the three methyl groups to generate phosphocholine, thus entering the CDP-choline pathway to synthesize PC. Based on our results, we hypothesize that other methylation enzymes in cells may play an important role in the methylation reaction for the PC synthesis in the absence of ChoC.

In summary, ChoC directly affects PC synthesis, thereby interfering with the metabolism of other lipids, such as PE, TAG, and DAG, damaging cell wall integrity, and weakening the pathogenicity in *A. fumigatus*. This study explored the phospholipid metabolic pathway in *A. fumigatus* and its relationship with growth and metabolism. ChoC, based on its relatively conserved characteristics in filamentous fungi and its special function for mammals, may be an excellent target for new antifungal drugs.

## MATERIALS AND METHODS

### Strains, growth conditions, and media

The strains used in this study are listed in Table 1. *A. fumigatus* 293.1 was the original WT strain used in the present study. Minimal medium was used as the basic medium, and supplements were added according to our experimental design. MM was supplemented with 0.1% yeast extract to harvest *A. fumigatus* spores. To explore the function of *choC* in the methylation pathway, 50 mg/L L-serine, 40 mg/L ethanolamine, 50 mg/L *N-*methylaminoethanol, 60 mg/L *N*, *N-*dimethylaminoethanol or 50 mg/ L choline chloride, 1.2 M sorbitol, and 2.5 mg/mL Congo red were added to the MM. For liquid-submerged culture, 1 × 10^6^ conidia/mL were inoculated into 100 mL liquid media and cultured at 37°C and 120 rpm. All strains were cultivated at 37°C. For the induction of asexual development, mycelia cultured in liquid MM medium at 37°C for 18 h were transferred to solid plates and cultured at 37°C as described (62). Photomicrographs were captured using an Eclipse 80i epifluorescence microscope (Nikon, Tokyo, Japan). Culture plates and northern blot images were obtained using a DSC-F707 digital camera (Sony, Tokyo, Japan).

**TABLE 1:** Strains used in this study

Strain	Genotype	Host
Δ*choC*	∆*choC*::*AnpyrG* +	*Afu293.1*
∆*choC*:: *choC*	∆*choC*::*AnpyrG* +; *choC*(p)::*choC*; *ptrA*(*pTR1*)	*Afu293.1*
∆*ck1*	∆*ck1*::*hyg*	*Afu293.1*
∆*ck2*	∆*ck2*::*hyg*	*Afu293.1*
Δ*choC* ∆*ck1*	∆*choC*::*pyrG*; ∆*ck1*::*hyg*	*Afu293.1*

### Determination of the *choC* ORF

Total RNA was extracted from mycelia cultured in liquid-merged medium for 48 h and reverse transcribed into cDNA (Thermo Fisher Scientific Inc., USA). Random primers for oligo-dT and cyclic reverse transcriptase were used in the reaction. To amplify cDNA through PCR reactions, we used primers c-*choC* S (upstream of the 5′ end of the *choC* ORF) and c-*choC* AS (downstream of the 3′ end of the *choC* ORF). Genomic DNA was amplified using *choC* S (upstream of the 5′ end of the *choC* ORF) and *choC* AS (downstream of the 3′ end of the *choC* ORF) primers. The intron sequence was verified by comparing the sequence of cDNA with the DNA sequence of the *choC* ORF.

### Cell dry weight and alamar blue assays

For the quantification of mycelial dry weight, the mycelia were harvested from the liquid medium during cultivation every alternate day. Samples were filtered through nitrocellulose filters (pore size 0.45 µm), washed with distilled water, and freeze-dried until constant weight was measured. Cell viability was measured as a percentage of reduction in alamar bluewith absorbance values determined at 570 and 600 nm. Detailed procedures and calculation methods have been described previously (26).

### Evans blue staining

Mycelia collected from the MM liquid medium at a certain time (37°C, 120 rpm) were treated with 0.1% Evans blue for 5 min at room temperature, washed three times with phosphate buffered saline (PBS), and then placed on slides for examination under bright-field illumination with an Eclipse 80i microscope (Nikon).

### Nucleic acid manipulation

For genomic DNA preparation, mycelia collected were ground into powder using liquid nitrogen, then treated with 500 µL of breaking buffer (8% sucrose, 5% Triton X-100, 50 mM EDTA, 50 mM Tris-Cl, pH 8.0) per 20 mg and incubated at 65°C for 30 min. This was followed by the addition of 500 µL of phenol:chloroform:isoamyl alcohol (25:24:1) to purify the genome. Isopropyl alcohol was added to precipitate the DNA at −20°C. The precipitate collected after centrifugation was resolved in 150 µL distilled water and treated with RNaseA. Genomic DNA was obtained following a second purification step. To isolate total RNA, 1 mL of TRIZOL reagent was added to each tube with 20 mg of mycelia powder for 10 min, followed by 200 µL of chloroform to purify the nucleic acids. Nucleic acids were precipitated by adding isopropyl alcohol at −20°C and treated with DnaseI (5 U/µL) to get rid of the genomic DNA. The mixture was treated once with phenol:chloroform:isoamyl alcohol (25:24:1) and then once with chloroform:isoamyl alcohol (24:1) until proteins, polysaccharides, and other impurities were eliminated. Finally, RNA was isolated by adding one-tenth volume of 3 M NaAc and twofold volume of absolute ethanol in a −80°C freezer for 20 min and resolved with RNase-free water for reverse transcription reactions.

Northern blot analysis was performed as described (63). For northern blotting, *choC* probes were designed using the genomic DNA of *A. fumigatus* 293.1 as a template. The probes were labeled with ^32^P-dCTP and used for hybridization in a modified Church buffer as previously described (64). About 10 µg of total RNA was separated by electrophoresis using a 1% agarose gel containing 6% probes. Primer pairs used in this study are listed in Table S4.

### Clustering and sequencing

Clustering of the index-coded samples was performed on a cBot Cluster Generation System using the TruSeq PE Cluster Kit v3-cBot-HS (Illumina, USA) according to the manufacturer’s instructions. After cluster generation, library preparations were sequenced on an Illumina HiSeq 2500 platform, and 125/150 bp paired-end reads were generated.

### Construction of Δ*choC* and *choC* complementation strains

DJ-PCR was used to construct the Δ*choC* mutant. The 5′-flanking and 3′-flanking regions of *choC* gene were amplified from the genomic DNA of *A. fumigstus* 293.1 with primer pairs oligo 3,995–3997 and oligo 3,996–3,998. *A. nidulans pyrG* gene was amplified from *A. nidulans* FGSC4 using the primer pair oligo 3,868–3,869. The third-round product of DJ-PCR was amplified using the primer pair oligo 3,999–4,000. The *A. fumigatus* DJ-PCR fragments were purified and used to transform protoplasts generated using the Vino Taste Pro lysis enzyme (65). A solid medium without uracil or uridine was used to select the *pyrG* + transformants. PCR and restriction enzyme digestion were used to verify the correct transformants. Three *A. fumigatus choC* deletion mutants were obtained. The *choC* gene was amplified from the genome of *A. fumigatus* 293.1 using the primer pairs 4,434–4,435. The *A. fumigatus choC* gene was cloned into pPTR1 with *Sma*I digestion. Protoplasts of a Δ*choC* strain were transformed with the recombinant pPTRI-*choC*. Solid MM with 1 µg/mL pyrithiamine was used to select the transformants. We used diagnostic PCR reactions to confirm the correct recombination of the plasmid. Finally, through functional phenotypic validation, three *A. fumigatus* Δ*choC::choC* complemented strains were obtained.

### Knockout of *CK1*, *CK2* in WT and Δ*choC* strains

The deletion vector pOSCAR-Gcn2 was constructed using the OSCAR protocol described previously (66). Approximately 1-kb fragments from upstream and downstream of the target genes *CK1/CK2* were amplified using PCR with the primer sets listed in Table S4, and *A*. *fumigatus* 293.1 genomic DNA as the template. The amplified fragments were assembled by introducing PCR products for each gene into the pA-Hyg-OSCAR marker vector and pOSCAR using the BP Clonase II enzyme (Invitrogen, Carlsbad, CA, USA). Then, the reaction mixture was transformed into *E. coli* DH5α. Bacterial colonies were obtained on LB plates with 100 µg/mL spectinomycin, following overnight incubation at 37°C. Disruption of *CK1/CK2* in *A. fumigatus* was achieved via *A. tumefaciens*-mediated genetic transformation. pOSCAR-CK1/pOSCAR-CK2 were transformed into *A. tumefaciens* LBA4404 using the protocol described previously (67). Then, the right transformants were cocultured with 10^7^ *A. fumigatus* 293.1 conidia at 37°C on a nitrocellulose ﬁlter that was spread on an IM plate supplemented with 100 mg/L hygromycin and 40 mg/L acetosyringone (Sigma, St. Louis, MO, USA). After induction for 2 days, the ﬁlter was transferred onto an MMY plate supplemented with cefotaxime (100 mg/L) followed by incubation for 2 days at 37°C for sporulation. Individual fungal transformants were obtained by single-spore isolation. Two pairs of primers, CK1-5′ Sense/Hygas and Hygs/CK1-3′ Anti-sense, were used to verify the construct of Δ*choC* strain.

### RNA-seq analysis

Clean data (clean reads) were obtained by removing reads containing adapters, poly-N, and low-quality reads from the raw data (raw reads) using in-house Perl scripts. Simultaneously, the Q20, Q30, and GC contents of the clean data were calculated. All downstream analyses were based on clean, high-quality data. The reference genome and gene model annotation files for *A. fumigatus* were downloaded directly from the website (http://ensemblgenomes.org/). A reference genome index was built using Bowtie v2.2.3, and clean paired-end reads were aligned to the reference genome using TopHat v2.0.12. HTSeq v0.6.1 was used to count the number of reads mapped to each gene. The fragments per kilobase per million base pairs sequenced of each gene were then calculated based on the length of the gene and the read count mapped to the gene.

Differential expression analysis of three biological replicates per sample was performed using the DESeq R package (version 1.18.0). The resulting *P*-values were adjusted using Benjamini and Hochberg’s approach to control the false discovery rate. Genes with an adjusted *P*-value < 0.05, found by DESeq, were considered differentially expressed. GO enrichment analysis of differentially expressed genes was performed using the GO seq R package, in which the gene length bias was corrected. GO terms with corrected *P*-values < 0.05 were considered significantly enriched by differentially expressed genes. We used the KOBAS software to test the statistical enrichment of differentially expressed genes in the KEGG pathways. The protein-protein interaction (PPI) analysis of differentially expressed genes was based on the STRING database, which contains known and predicted protein-protein interactions. For species existing in the database, we constructed networks by extracting the target gene list from the database; otherwise, Blastx (v2.2.28) was used to align the target gene sequences to the selected reference protein sequences, and then the networks were built according to the known interactions of the selected reference species.

### Lipid extraction

A certain amount of hyphae of each sample (30 mg) was mixed with acetonitrile, and the mixture was kept at 4°C for 4 h. A quantity of 200 µL of extract was taken, and 500 µL of CHCl_3_-CH_3_OH (2:1, vol/vol) was added, vortexed, and 200 µL of water was subsequently added. The mixture was centrifuged to remove the organic layer, dried, and resolved with 200 µL of acetonitrile-isopropanol for testing.

### High performance liquid chromatography-mass spectrometry (HPLC-MS)

Chromatography was performed on an ACQUITY UPLC-Xevo G2 Q-TOF UPLC system (Waters, USA). Briefly, the compounds were eluted from a UPLC BEHC18 column (Waters, 1.7 µm, 2.1 × 100 mm) in an isocratic gradient consisting of 60% (A) ultrapure water/acetonitrile (4:6, vol/vol) and 40% (B) acetonitrile/isopropanol (1:9, vol/vol) containing 0.1% formic acid and 5 mM acetamide as mobile phases. The flow rate and column temperature were set at 0.3 mL/min and 55°C, respectively. The LC system was coupled to a Q-TOF tandem mass spectrometer fitted with an electrospray ionization (ESI) source. The ESI ion source has a mass scanning range of 50–1,200 *m/z*. Nitrogen was used for all gas paths. The operating parameters are listed in Table 2. The compounds were monitored in both positive and negative ion modes. The software package Masslynx 4.1 was used for mass spectral data acquisition and quantitation.

**TABLE 2:** Operating parameters of mass spectrometry

Survey ion mode and polarity	+ESI	−ESI
Mass (*m/z*)	50–1,200	50–1,200
Capillary (V)	2,500	−2,500
Sampling cone (V)	35	−35
Desolvation temperature (°C)	350	350
Desolvation gas flow (L/H)	700	700
Cone gas flow (L/H)	50	50
Source temperature (°C)	400	400

### Data processing

Conversion of mass spectrometry data was carried out using MZmine software after the following steps: denoising, mass spectral peak extraction, deconvolution processing, peak arrangement, alignment, merging, listing in Excel and re-denoising, gap filling, data export, and other processes. The final data format included ID, *m/z*, RT, and (sample number) integral area (the original data can be found in Tables S1 to S8). Data analysis could be performed using raw or normalized processed data.

### Survival and fungal burden tests

BALB/c mice were given free access to water and food and were maintained in a temperature-controlled (22°C) room with a 12/12 h light/dark cycle (8:00 a.m., lights on; 8:00 p.m., lights off). Mice received an intravenous injection of 0.1 mL of the inocula and were monitored for 3 weeks after the injection. The mortality rate was recorded daily. Liver, spleen, and kidney tissues from mice infected with different strains were removed and homogenized with an appropriate volume of 1× PBS. Primary homogenate dilutions were quantitatively cultured by serial dilution, plated on MMY plates, and incubated at 37°C for 24–36 h, after which *A. fumigatus* fungal burdens (numbers of CFU per gram of lung tissue) were determined.

### Histopathological study

Infected animals were sacrificed on days 3, 13, and 23 after inoculation. The lungs, liver, spleen, and kidneys were fixed in formalin, and paraffin-embedded sections were stained with HE and PAS and photographed under a light microscope.

### Data analysis

All experiments were randomized, and the scientists who performed the quantification were blinded. Statistical analyses were performed using analysis of variance (ANOVA) and Student’s *t*-test, unless specified otherwise, using GraphPad software. Student’s *t*-tests were used to compare the two conditions. A one-way ANOVA was used to compare multiple experimental conditions. The Bonferroni *post hoc* test was used to compare each condition. For dry weight and alamar blue reduction analysis, a two-way ANOVA was used for comparisons at different time points. All data are shown as the mean ± SEM. Statistical significance was set at *P* < 0.05 significant.

## Data Availability

The data sets presented in this study can be found in online repositories. The RNA-seq data has been uploaded to NCBI (https://www.ncbi.nlm.nih.gov/bioproject/PRJNA471263).

## SUPPLEMENTAL MATERIAL

Fig. S1 to S5 and Tables S1 to S8 spectrum.02168-23-s0001.pdf Description of supplementary information on different gene expression levels, strain growth morphology, PC content, etc.

ASM does not own the copyrights to Supplemental Material that may be linked to, or accessed through, an article. The authors have granted ASM a non-exclusive, world-wide license to publish the Supplemental Material files. Please contact the corresponding author directly for reuse.
