# Supplementary material for: Functional, transcriptomic, and lipidomic studies of the choC gene encoding a phospholipid methyltransferase in Aspergillus fumigatus
Source: Microbiol Spectr. 2023 Nov 27;12(1):e02168-23. doi: 10.1128/spectrum.02168-23 (PMC10783049; doi:10.1128/spectrum.02168-23)
Supplement: Fig. S1 to S5 and Tables S1 to S8 — Description of supplementary information on different gene expression levels, strain growth morphology, PC content, etc. [file spectrum.02168-23-s0001.pdf]

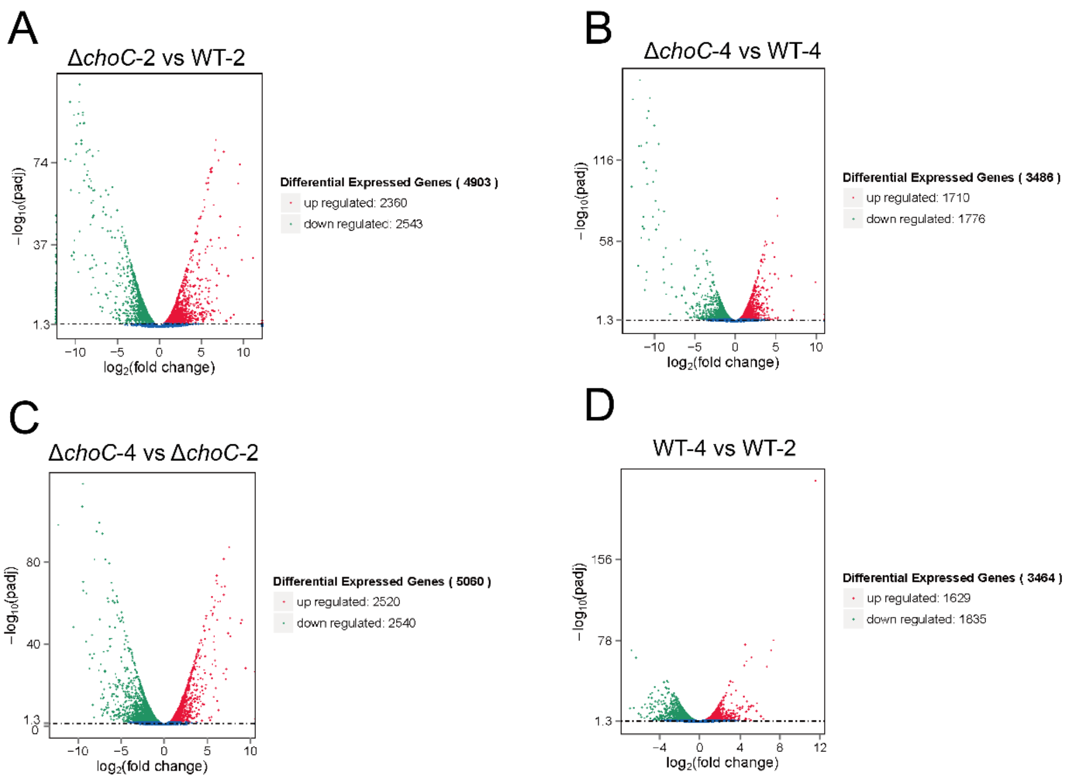

Figure S1.There are different expressed genes between *ΔchoC* and *Afu-WT* strains (A) Between Δ*choC*-2 and WT-2, there are 2360 up-regulated genes and 2543 down-regulated genes. (B) Between Δ*choC*-4 and WT-4, there are 1710 up regulated genes and 1776 down regulated genes; (C) Between Δ*choC* -4 and Δ*choC* -2, there are 2520 up regulated genes and 2540 down regulated genes; (D) Between WT-4 and WT-2, there are 1629 up regulated genes and 1835 down regulated genes. Red is symbol of up-regulated, green is symbol of down- regulated.

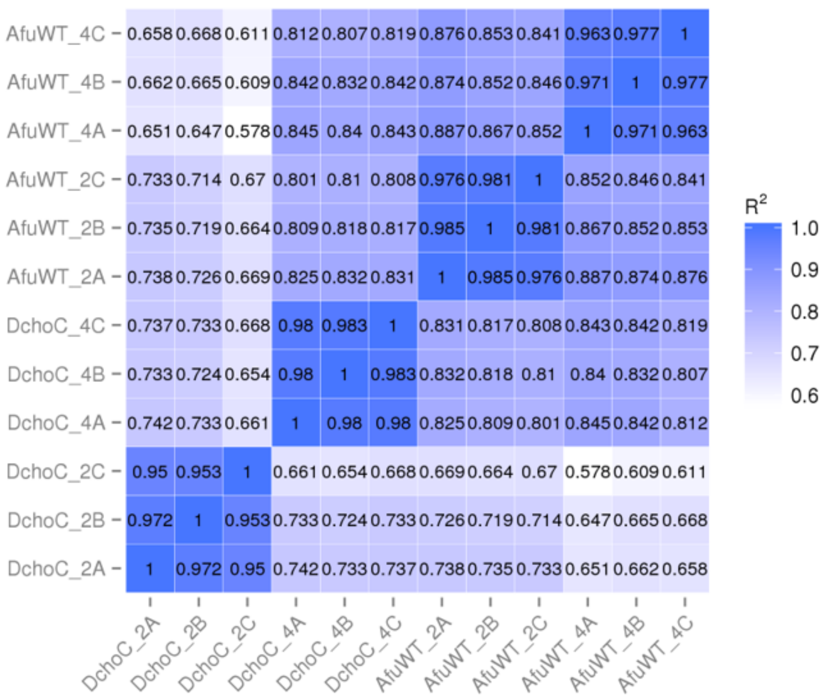

Figure S2. Pearson correlation between samples.

In project operations, we require that the square of the pearson (R^2) between biological replicates be at least greater than 0.8.The result showed good repeatability among the triplicates.

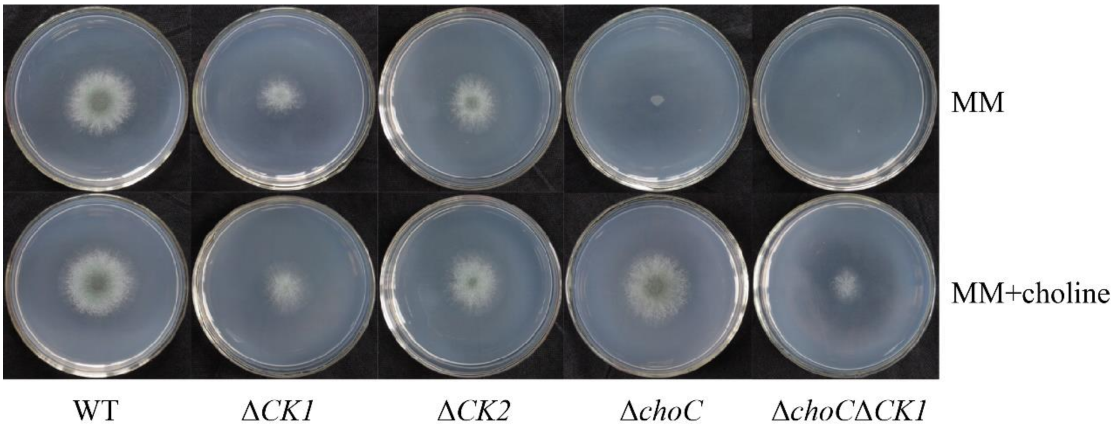

Figure S3. Effect of *CK1* and *CK2* on the growth of strains.

The colony morphology of five different types WT、Δ*choC*、Δ*ck1*、Δ*ck2*、Δ*choC*Δ*ck1* on solid plate MM and MM+choline

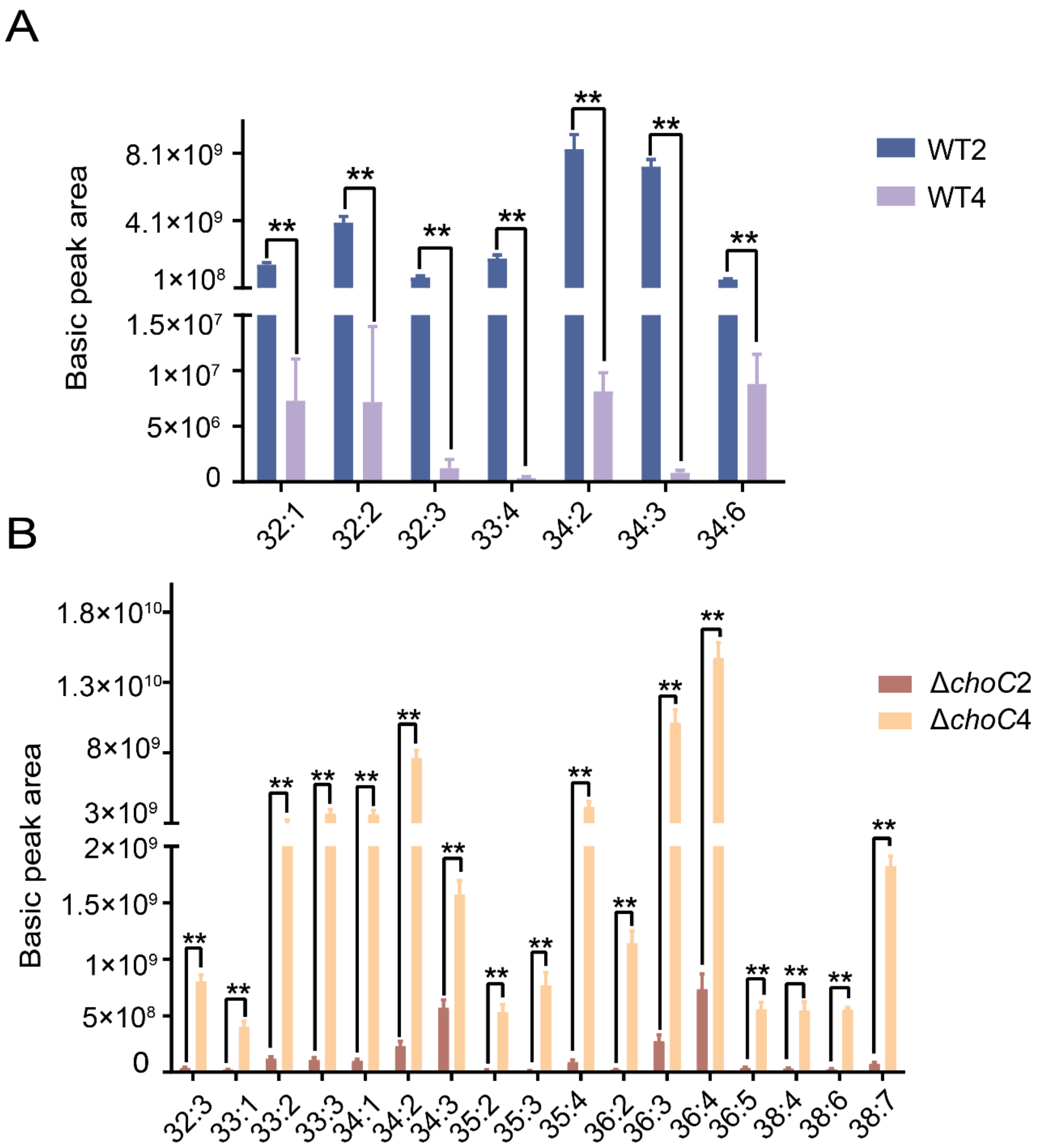

Figure S4. The content of PCs in the wild type strain and Δ*choC* mutant strain

(A) Abundance of PC in the wild-type strain at day 2 and day 4. (B) Abundance of PC in the *ΔchoC* mutant strain at day 2 and day 4. N=6, $*p < 0.05$; $**p < 0.01$; $***p < 0.001$

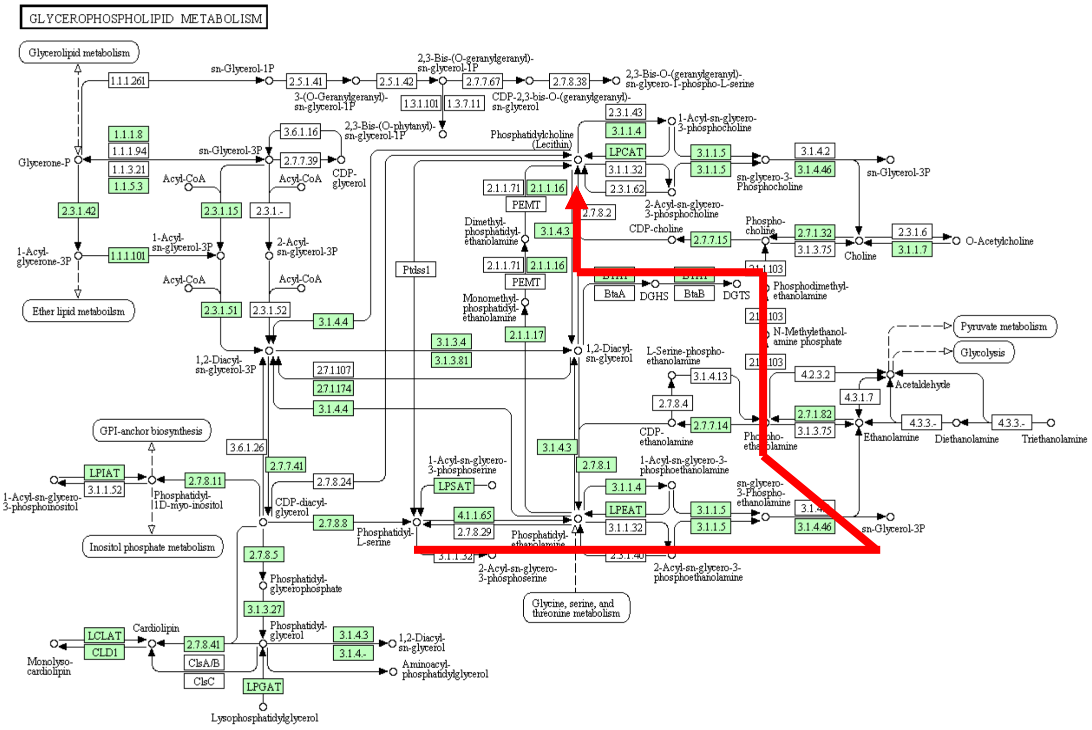

Figure S5. Part of the glycerophospholipid metabolism in Δ*choC* strain at day 2. Our results indicate another pathway for the synthesis of PC (as shown by the red curve), where phosphoethanolamine undergoes a three-step methylation reaction to generate phosphocholine, which then leads to the CDP-choline pathway for the synthesis of PC.

Table S1. List of samples for transcriptomic analysis.

Comparative analysis	process	reference
**Group 1**	Δ*choC* -2	WT-2
**Group 2**	Δ*choC*-4	WT-4
**Group 3**	Δ*choC*-4	Δ*choC*-2
**Group 4**	WT-4	WT-2

Δ*choC*-2, Δ*choC*-4 mean Δ*choC* mutant strain were cultured in liquid MM for 2 days and 4 days, respectively; WT-2, WT-4 mean the wild type strain were cultured in liquid MM for 2 days and 4 days, respectively.

Table S2. Information of tested samples for lipidomic analysis.

Group	Number	Order	Weight
**WT2**	7	WT2 (A-G)	40mg
**WT4**	6	WT (A-F)	40mg
**Δ*choC*2**	6	Δ*choC*(A-F)	40mg
**Δ*choC*4**	6	Δ*choC*(A-F)	40mg

TableS3. Information of differential genes in heatmap

Gene ID	Gene Description	Gene	Pathway
CADAFUAG00008884	Choline kinase	*CK*	Glycerophospholipid metabolism
CADAFUAG00007631	PS decarboxylase	*ChoB*	
CADAFUAG00007260	Ethanolamine kinase	*EK*	
CADAFUAG00008266	Patatin family phospholipase phospholipase A2	*Pla2*	
CADAFUAG00007510	CDP-diacylglycerol-inositol 3-phosphatidyltransferase PIS	*PIS*	Phosphotidylinositol signaling system
CADAFUAG00005925	Protein kinase c	*Pkc*	
CADAFUAG00007273	PI phospholipase C	*Plc1*	
CADAFUAG00007745	Calmodulin	*Calm*	
CADAFUAG00007699	Inositol monophosphatase QutG	*QutG*	
CADAFUAG00008714	Transcription factor AbaA	*AbaA*	MAPK signaling system
CADAFUAG00006197	A-pheromone receptor	*PreA*	

CADAFUAG00001922	Ceramide synthase membrane component	*LAG1*	Sphingolipid metabolism
CADAFUAG00006951	Serine palmitoyltransferase	*SPT*	
CADAFUAG00008210	Dihydroceramide desaturase	*DEGS*	
CADAFUAG00005966	Autophagy-related protein 17 kinase activator	*ATG17*	Autophagy
CADAFUAG00003192	Vacuolar protein sorting 30	*VPS30*	
CADAFUAG00006382	Autophagocytosis protein ubiquitin-like-conjugating enzyme	*ATG3*	
CADAFUAG00001732	Autophagocytosis protein ubiquitin-like-conjugating enzyme	*ATG12*	
CADAFUAG00007423	Vacuolar ATP synthase subunit H	*V-ATPase*	Oxidative phosphorylation(Phagosome)
CADAFUAG00000698	ATP synthase subunit E	*ATPase*	
CADAFUAG00002825	DNA replication licensing factor Mcm5	*Mcm5*	Cell cycle
CADAFUAG00009054	DNA replication licensing factor Mcm3	*Mcm3*	
CADAFUAG00004775	DNA replication licensing factor Mcm3	*Mcm4*	
CADAFUAG00001771	Nuclear pore complex subunit	*Bub3*	
CADAFUAG00007568	Cell division cycle protein	*Cdc20*	
CADAFUAG00001802	DASH complex component	*Dam1*	

Table S4. Oligonucleotides used in this study

Name	Sequence	Description
oligo3868	AGCAATGTAAAGCTAACGTGCGTG	Forward primer for Ani-pyrG
oligo3869	TTTGCCTTTAAGCTTCGGGTAGAG	Reverse primer for Ani-pyrG
oligo3995	CTGAAACGTCTGGCATCGGATA	*choC* deletion 5'-forward
oligo3996	GGTGACGAGCTTGATGAGGATG	*choC* deletion 3'-reverse
oligo3997	TTGGGCTCACGCACGTTAGCTTTAC CCTACAATGCTGGTCAGTTGTGAA	*choC* deletion 3'-reverse with complementary 5'-*pyrG* tail
oligo3998	CACATACTCTACCCGAAGCTTAAAG AAGCAACACACCGCTTACGTGCAG	*choC* deletion 5'-forward with complementary 3'-*pyrG* tail
oligo3999	GAGCGGTCAGGATCGGAAAGATA	*choC* deletion construct 5'-nest
oligo4000	GGTGGATGCTCCAAATTGCGAG	*choC* deletion construct 3'-nest
oligo4434	CCTCGGGTTTACTCGAAGAACT	Afu-Δ*choC* Whole sequence 5'forward
oligo4435	GAACCCGGGCTGTGCTACCTGATCCC AACTT	Afu-Δ*choC* Whole sequence 3'Forward
oligo4436	ATGTGCTCTCTTCATCTTGTTC	Afu-choC probe 5'-forward
oligo4437	TTACAAGCTCTTGCCGCCCT	Afu-choC probe 3'-reverse
Afu-cDNA-choC S	CTCTTCATCTTGTTCAATCCAC	*choC* ORF 5'-forward for Afu cDNA
Afu-cDNA-choC AS	CGTTCCCGTTCACGCTTA	*choC* ORF 3'-reverse for Afu cDNA
Afu-genome-choC S	CGTTGTCTCATTCTTGACCCAT	*choC* ORF 5'-forward for Afu genome
Afu-genome-choC AS	CCTTTCTCCCTCTATCACC	*choC* ORF 3'-reverse for Afu genome
CK1-5' Sense	GGGGACAGCTTTCTTGTACAAAGTGG AAGGCGATTGAAGGATAAGG	5'-forward primer for *Afu ck1*
CK1-5' Anti-sense	GGGGACTGCTTTTTTGTACAAACTTG TATGGGACACCACAAACAAAT	5'-reverse primer for *Afu ck1*
CK1-3' Sense	GGGGACAACTTTGTATAGAAAAGTTG TTGCTCCAACAACTCCACATC	3'-forward primer for *Afu ck1*
CK1-3' Anti-sense	GGGGACAACTTTGTATAATAAAGTTG TGACGGCACCTACATCACC	3'-reverse primer for *Afu ck1*
CK2-5' Sense	GGGGACAGCTTTCTTGTACAAAGTGG AAGGCGATTGAAGGATAAGG	5'-forward primer for *Afu ck2*
CK2-5' Anti-sense	GGGGACTGCTTTTTTGTACAAACTTG TATGGGACACCACAAACAAAT	5'-reverse primer for *Afu ck2*
CK2-3' Sense	GGGGACAACTTTGTATAGAAAAGTTG TTGCTCCAACAACTCCACATC	3'-forward primer for *Afu ck2*
CK2-3' Anti-sense	GGGGACAACTTTGTATAATAAAGTTG TGACGGCACCTACATCACC	3'-reverse primer for *Afu ck2*
CK1-up Sense	GCCGAGGAGACAGTAACCC	*ck1* forward probe for sequencing
CK1-down Antisense	TGCATCGCATTCATACCC	*ck1* reverse probe for sequencing
Hygs	GCCCTTCCTCCCTTTATT	*hyg* forward probe for sequencing
Hygas	ACTGGCAAACTGTGATGGA	*hyg* reverse probe for sequencing
CK2-up Sense	TGGAAGCATCGGAGATAGCG	*ck2* forward probe for sequencing
CK2-down Antisense	GCATCCCAGCCAAACATACC	*ck2* reverse probe for sequencing

Table S5. Differential gene KEGG enrichment analysis of Δ*choC-2* vs. WT-2.

Gene ID	Description	Regulation	log_2FC	p-value
**Oxidative phosphorylation**				

CADAFUAG00004559	NADH-ubiquinone dehydrogenase 24 kDa subunit	+	1.1037	5.94×10^{-5}
CADAFUAG00002749	NADH-ubiquinone oxidoreductase	+	1.395	3.71×10^{-7}
CADAFUAG00001945	NADH-ubiquinone oxidoreductase 304 kDa subunit	+	1.2378	7.53×10^{-6}
CADAFUAG00007123	NADH-ubiquinone oxidoreductase 19 kDa subunit	+	1.2751	3.59×10^{-6}
CADAFUAG00000468	NADH-ubiquinone oxidoreductase 105 kDa subunit	+	1.317	5.41×10^{-3}
CADAFUAG00004559	NADH-ubiquinone dehydrogenase 24 kDa subunit	+	1.1037	5.94×10^{-5}
CADAFUAG00002749	NADH-ubiquinone oxidoreductase	+	1.395	3.71×10^{-7}
CADAFUAG00005921	Iron-sulfur protein subunit of succinate dehydrogenase Sdh2	-	1.1693	7.85×10^{-6}
CADAFUAG00005718	Succinate dehydrogenase subunit Sdh1	-	1.0318	6.67×10^{-5}
CADAFUAG00001607	Inorganic diphosphatase	-	2.1118	2.08×10^{-11}
CADAFUAG00006375	Cytochrome c oxidase subunit V	+	1.597	4.34×10^{-9}
CADAFUAG00005595	Cytochrome c oxidase subunit 7A	+	1.5261	2.13×10^{-8}
CADAFUAG00005824	Cytochrome c oxidase subunit VIa	+	1.7458	1.56×10^{-10}
**Glycerophospholipid metabolism**				
CADAFUAG00004565	Phospholipase D (PLD)	+	1.1239	2.45×10^{-5}
CADAFUAG00005036	Phospholipase D1 (PLD1)	-	1.0889	5.51×10^{-5}
CADAFUAG00008838	Phosphatidate cytidylyltransferase	-	1.1088	4.81×10^{-5}
CADAFUAG00007510	CDP-diacylglycerol-inositol 3-phosphatidyltransferase PIS	-	1.5167	5.48×10^{-8}
CADAFUAG00003739	Glycerophosphoryl diester phosphodiesterase family protein phosphatidylglycerol phospholipase C	+	3.4764	7.80×10^{-6}
CADAFUAG00007261	Phospholipid methyltransferase	+	1.2429	8.54×10^{-6}
CADAFUAG00007631	Phosphatidylserine decarboxylase	+	4.8192	1.19×10^{-20}
CADAFUAG00008266	Patatin family phospholipase phospholipase A2 (PlaA)	+	1.1867	9.31×10^{-4}
CADAFUAG00008884	Choline kinase		**-Inf**	1.08×10^{-12}
CADAFUAG00007260	Ethanolamine kinase	+	1.8069	1.23×10^{-10}
CADAFUAG00008541	Phosphoethanolamine ethanolamine-phosphate cytidylyltransferase	+	1.8254	1.86×10^{-10}
CADAFUAG00002978	sn-1,2-diacylglycerol cholinephosphotransferase	-	1.2069	1.36×10^{-5}
**MAPK signaling pathway**				

CADAFUAG00006197	A-pheromone receptor PreA	-	2.7184	1.37×10^{-20}
CADAFUAG00003326	Serine/threonine-protein kinase ste20	-	1.0495	1.90×10^{-4}
CADAFUAG00006950	MAP kinase kinase kinase SskB	-	1.2195	1.59×10^{-5}
CADAFUAG00008714	Transcription factor AbaA	-	2.4262	5.61×10^{-8}
CADAFUAG00002199	Lactoylglutathione lyase	+	1.5282	2.37×10^{-8}
	**Phosphotidylinnositol signaling system**			
CADAFUAG00007273	1-phosphatidylinositol-4,5-bisphosphate phosphodiesterase PLC1	+	0.89582	1.88×10^{-2}
CADAFUAG00007699	Inositol monophosphatase QutG	-	1.838	3.15×10^{-7}
CADAFUAG00008838	Phosphatidate cytidylyltransferase	-	1.1088	4.81×10^{-5}
CADAFUAG00005925	Protein kinase c	-	0.65339	1.34×10^{-2}
CADAFUAG00007510	CDP-diacylglycerol-inositol 3-phosphatidyltransferase PIS	-	1.5167	5.48×10^{-8}
CADAFUAG00007745	Calmodulin	+	1.7954	7.28×10^{-4}
**Meiosis yeast**				
CADAFUAG00004157	MFS monosaccharide transporter	+	6.8455	7.12×10^{-28}
CADAFUAG00004724	APSES transcription factor StuA	-	1.7271	1.71×10^{-10}
CADAFUAG00004775	DNA replication licensing factor Mcm4	-	1.3004	1.51×10^{-6}
CADAFUAG00005114	DNA replication licensing factor Mcm2	-	1.1258	3.98×10^{-5}
CADAFUAG00005968	DNA replication licensing factor Mcm6	-	1.4648	1.27×10^{-7}
CADAFUAG00009054	DNA replication licensing factor Mcm3	-	1.2274	9.69×10^{-6}
CADAFUAG00002825	DNA replication licensing factor Mcm5	-	1.9275	1.18×10^{-11}
CADAFUAG00006846	Origin recognition complex subunit Orc1	-	1.1885	5.68×10^{-5}
CADAFUAG00004390	NDT80 / PhoG like DNA-binding family protein meiosis-specific transcription factor NDT80	-	1.1289	3.94×10^{-5}
CADAFUAG00008947	Serine/threonine-protein phosphatase PP1 catalytic subunit	-	1.0239	1.42×10^{-4}
CADAFUAG00003650	Serine/threonine-protein phosphatase PP1 catalytic subunit	-	1.1795	1.10×10^{-5}
CADAFUAG00007568	Cell division cycle protein Cdc20	+	1.1158	2.02×10^{-4}
	**Regulation of Autophagy**			
CADAFUAG00004709	autophagy-related protein 17 kinase activator (Atg17)	+	1.638	9.23×10^{-6}
CADAFUAG00003192	vacuolar armadillo repeat protein Vac8	+	2.1025	7.13×10^{-10}
CADAFUAG00006382	autophagocytosis protein Aut1 ubiquitin-like-conjugating enzyme ATG3	+	1.0788	5.21×10^{-3}

	**Phagosome**			
CADAFUAG00005157	Vacuolar ATP synthase 16 kDa proteolipid subunit, putative	+	1.1097	3.58×10^{-5}
CADAFUAG00007371	Vacuolar ATP synthase subunit c	+	1.0756	7.00×10^{-3}
CADAFUAG00007423	Vacuolar ATP synthase subunit H	+	1.6165	3.37×10^{-9}
CADAFUAG00000698	ATP synthase subunit E	+	1.4445	1.18×10^{-7}
CADAFUAG00001064	Tubulin beta chain	+	1.2403	6.46×10^{-6}
CADAFUAG00005223	Protein translocation complex subunit Sss1	+	1.1895	5.89×10^{-4}
CADAFUAG00002303	NADPH oxidase (NoxA)	+	1.246	2.65×10^{-3}
**Ubiquitin mediated proteolysis**				
CADAFUAG00005443	Ubiquitin conjugating enzyme (UbcB)	+	1.5211	1.91×10^{-3}
CADAFUAG00004384	Ubiquitin conjugating enzyme	+	2.2817	8.20×10^{-15}
CADAFUAG00003920	U-box domain protein	+	2.2851	1.32×10^{-8}
CADAFUAG00004971	F-box and WD40 domain protein	-	3.514	1.59×10^{-32}
CADAFUAG00002258	Transcriptional elongation regulator Elc1/Elongin C	-	1.5621	6.28×10^{-8}
**Protein processing in ER**				
CADAFUAG00005223	Protein translocation complex subunit Sss1	+	1.1895	5.89×10^{-4}
CADAFUAG00007667	Probable mannosyl-oligosaccharide alpha-1,2-mannosidase 1B	-	1.4501	7.79×10^{-8}
CADAFUAG00001600	Class I alpha-mannosidase 1A	-	1.6799	1.33×10^{-9}
CADAFUAG00004876	DnaJ domain protein (Mas5)	-	1.4292	1.57×10^{-7}
CADAFUAG00000734	ER-associated proteolytic system protein Der1	+	1.0838	1.11×10^{-4}
CADAFUAG00006765	Heat shock protein Hsp20/Hsp26	+	4.7115	1.85×10^{-19}
CADAFUAG00006211	Polyubiquitin binding protein (Doa1/Ufd3)	+	1.3204	1.60×10^{-6}
CADAFUAG00002351	Ubiquitin-like protein DskB	+	1.3242	1.66×10^{-6}
CADAFUAG00004708	Ubiquitin conjugating enzyme (UbcF)	+	1.1188	5.43×10^{-5}
CADAFUAG00002784	Ubiquitin conjugating enzyme (UbcH)	+	1.4896	3.81×10^{-4}
CADAFUAG00004773	AMFR protein	+	1.4388	1.03×10^{-4}
CADAFUAG00002784	Ubiquitin conjugating enzyme (UbcH)	+	1.4896	3.81×10^{-4}
CADAFUAG00005443	Ubiquitin conjugating enzyme (UbcB)	+	1.5211	1.91×10^{-3}
**Snare interactions in vesicular transport**				
CADAFUAG00007858	SNARE complex subunit (Tlg2)	+	1.353	6.14×10^{-3}

CADAFUAG00001889	Synaptobrevin-like protein Sybl1	+	1.4122	$4.72×10^{-7}$
CADAFUAG00005547	ER-Golgi SNARE complex subunit (Sed5)	+	0.64407	$1.41×10^{-2}$
CADAFUAG00006867	Golgi SNAP receptor complex member 1	+	0.80494	$2.92×10^{-3}$
CADAFUAG00008731	SNARE complex subunit (Bet1)	+	0.65185	$1.81×10^{-2}$
CADAFUAG00003920	U-box domain protein	+	2.2851	$1.32×10^{-8}$
**Proteasome**				
CADAFUAG00009176	Proteasome regulatory particle subunit (RpnF)	+	1.8466	$7.86×10^{-4}$
CADAFUAG00002328	Proteasome regulatory particle subunit (RpnI)	+	1.6198	$5.58×10^{-9}$
CADAFUAG00005585	Proteasome component Prs2	+	1.8229	$5.79×10^{-3}$
CADAFUAG00009519	Proteasome subunit alpha type	+	1.9899	$1.18×10^{-7}$
CADAFUAG00002607	Proteasome component Pre6	+	1.5288	$2.69×10^{-8}$
CADAFUAG00004270	Proteasome component Pup2	+	2.0007	$5.68×10^{-3}$
CADAFUAG00000612	Proteasome subunit alpha type	+	1.5651	$8.69×10^{-7}$
CADAFUAG00009664	Proteasome subunit beta type	+	1.7925	$9.45×10^{-4}$
CADAFUAG00003899	Proteasome subunit beta type	+	1.5507	$2.05×10^{-8}$
**Cell cycle**				
CADAFUAG00007568	Cell division cycle protein Cdc20	+	1.1158	$2.02×10^{-4}$
CADAFUAG00002825	DNA replication licensing factor Mcm5	-	1.9275	$1.18×10^{-11}$
CADAFUAG00005114	DNA replication licensing factor Mcm2	-	1.1258	$3.98×10^{-5}$
CADAFUAG00005968	DNA replication licensing factor Mcm6	-	1.4648	$1.27×10^{-7}$
CADAFUAG00004775	DNA replication licensing factor Mcm4	-	1.3004	$1.51×10^{-6}$
CADAFUAG00009054	DNA replication licensing factor Mcm3	-	1.2274	$9.69×10^{-6}$
CADAFUAG00006846	Origin recognition complex subunit Orc1	-	1.1885	$5.68×10^{-5}$
CADAFUAG00001710	Cell division control protein Cdc4	-	1.415	$2.73×10^{-7}$
CADAFUAG00003105	Cell cycle regulatory protein Cks/Suc1	-	1.2252	$9.97×10^{-6}$
CADAFUAG00005067	Checkpoint protein kinase	+	1.0962	$6.93×10^{-5}$
CADAFUAG00001802	DASH complex component Dam1	+	1.2001	$1.12×10^{-3}$
CADAFUAG00001771	Nuclear pore complex subunit	+	1.4124	$3.84×10^{-7}$
CADAFUAG00001706	GTP binding protein (SPG1)	+	1.0111	$2.24×10^{-4}$

Note: The level of differences was compared between the mutant Δ*choC* and WT at day 2 for each gene by t-test. The difference level for each selected gene at $P < 0.05$. +, upregulated; -, downregulated. $Log_2FC \geq 1$, the difference in the transcription level of the selected genes is significant.

Table S6. Differential gene KEGG enrichment analysis of Δ*choC*-4 vs. WT-4.

GeneID	Gene description	Regulation	log_2FC	pval
**MAPK signaling pathway**				
CADAFUAG00008192	Phosphotransmitter protein Ypd1, putative	+	2.8818	3.93×10^{-38}
CADAFUAG00007322	MAP kinase kinase (Pbs2), putative	+	1.3251	4.06×10^{-10}
CADAFUAG00006975	Mitogen-activated protein kinase hog1	+	1.8229	1.04×10^{-17}
Phosphotidylinnositol signaling system				
CADAFUAG00007273	1-phosphatidylinositol-4,5-bisphosphate phosphodiesterase Plc1, putative	-	1.3634	3.86×10^{-8}
CADAFUAG00007699	Inositol monophosphatase QutG, putative	+	2.5687	7.38×10^{-27}
Regulation of Autophagy				
CADAFUAG00001732	Ubiquitin-like protein ATG12	+	1.4897	1.84×10^{-7}
**Phagosome**				
CADAFUAG00005938	Protein transport protein Sec61 alpha subunit, putative	+	1.0861	2.65×10^{-7}
**Ubiquitin mediated proteolysis**				
CADAFUAG00004384	Ubiquitin conjugating enzyme, putative	+	1.2036	2.32×10^{-3}
CADAFUAG00002258	Transcriptional elongation regulator Elc1/Elongin C, putative	-	1.6043	2.33×10^{-6}
**Protein processing in ER**				
CADAFUAG00007667	Probable mannosyl-oligosaccharide alpha-1,2-mannosidase 1B	+	2.2298	8.85×10^{-25}
CADAFUAG00008108	Class I alpha-mannosidase	+	1.484	1.06×10^{-11}
CADAFUAG00009275	Disulfide isomerase, putative	-	1.5477	7.98×10^{-7}
CADAFUAG00005938	Protein transport protein Sec61 alpha subunit, putative	+	1.0861	2.65×10^{-7}
CADAFUAG00000734	ER-associated proteolytic system protein Der1, putative	+	1.4039	2.34×10^{-9}
CADAFUAG00009111	Protein mitochondrial targeting protein (Mas1), putative	-	1.3697	2.17×10^{-10}
CADAFUAG00002754	Heat shock protein 90	-	1.6763	1.56×10^{-3}
**Peroxisome**				
CADAFUAG00003215	Peroxisomal membrane protein (PmpP24), putative	+	1.2187	5.55×10^{-8}
CADAFUAG00000630	Catalase A	+	2.0114	4.43×10^{-10}
CADAFUAG00000317	Catalase B	+	2.0274	3.86×10^{-21}
CADAFUAG00007892	Mevalonate kinase	-	1.3291	7.71×10^{-4}
**Meiosis yeast**				
CADAFUAG00004297	Protein kinase, putative	+	1.0466	1.27×10^{-5}
CADAFUAG00002777	Meiosis-specific topoisomerase Spo11, putative	+	2.4289	3.38×10^{-18}
**Cell cycle**				
CADAFUAG00002027	Cell cycle control protein tyrosine phosphatase	+	1.4452	8.29×10^{-11}

	Mih1, putative			
CADAFUAG00004297	Protein kinase, putative	+	1.0466	1.27×10^{-5}
**Glycerophospholipid metabolism**				
CADAFUAG00004416	Glycerol 3-phosphate dehydrogenase (GfdB), putative	+	1.707	1.05×10^{-6}
CADAFUAG00004565	Phospholipase D (PLD), putative	-	1.1346	3.43×10^{-4}
CADAFUAG00009512	Phospholipase PldA, putative	+	3.4129	1.87×10^{-27}
CADAFUAG00000092	Phosphatidylglycerol specific phospholipase, putative	+	1.3217	2.83×10^{-10}
CADAFUAG00008266	Patatin family phospholipase, putative	+	1.1209	4.42×10^{-6}
CADAFUAG00002626	Lysophospholipase 2	-	3.5909	3.22×10^{-9}
CADAFUAG00008884	Choline kinase, putative	-	**Inf**	1.83×10^{-20}
CADAFUAG00007575	Choline kinase, putative	+	1.0005	1.49×10^{-4}
CADAFUAG00007631	Phosphatidylserine decarboxylase, putative	+	2.8595	1.63×10^{-4}
CADAFUAG00008918	Alpha/beta hydrolase, putative	-	1.0707	2.52×10^{-4}
**Glycosphingolipid biosynthesis**				
CADAFUAG00002838	Probable alpha-galactosidase B	-	1.0813	1.39×10^{-5}
CADAFUAG00001015	Melibiase subfamily, putative	-	1.8466	1.69×10^{-9}
CADAFUAG00003685	Beta-N-hexosaminidase, putative	+	1.3092	3.82×10^{-9}
**Glycerolipid metabolism**				
CADAFUAG00001367	Putative uncharacterized protein	+	2.2696	5.98×10^{-16}
CADAFUAG00004295	Glycerol dehydrogenase, putative	+	1.2696	7.59×10^{-8}
CADAFUAG00008266	Patatin family phospholipase, putative	+	1.1209	4.42×10^{-6}
CADAFUAG00001015	Melibiase subfamily, putative	-	1.8466	1.69×10^{-9}
CADAFUAG00002838	Probable alpha-galactosidase B	-	1.0813	1.39×10^{-5}
CADAFUAG00002680	Extracellular lipase, putative	+	1.1929	1.28×10^{-7}
CADAFUAG00003816	Alkaline lipase, putative	+	2.0176	7.91×10^{-3}
CADAFUAG00001112	Extracellular lipase, putative	-	1.5562	1.33×10^{-2}
**Sphingolipid metabolism**				
CADAFUAG00008112	Longevity-assurance protein (LAC1), putative	+	1.179	3.09×10^{-7}
CADAFUAG00008744	Neutral/alkaline nonlysosomal ceramidase, putative	+	1.8867	2.42×10^{-17}
CADAFUAG00006390	Cell-associated beta-galactosidase, putative	+	1.4903	1.22×10^{-2}
CADAFUAG00003262	Beta-1,6-glucanase Neg1, putative	+	1.7122	2.40×10^{-4}
CADAFUAG00006736	Arylsulfatase, putative	+	1.5827	1.01×10^{-12}
CADAFUAG00001015	Melibiase subfamily, putative	-	1.8466	1.69×10^{-9}
CADAFUAG00002838	Probable alpha-galactosidase B	-	1.0813	1.39×10^{-5}
**Oxidative phosphorylation**				
CADAFUAG00003878	NADH-ubiquinone oxidoreductase 64 kDa subunit, putative	+	1.9719	3.22×10^{-20}
CADAFUAG00008495	Cytochrome b-c1 complex subunit 7	-	-1.1348	4.88×10^{-3}

CADAFUAG00007399	Ubiquinol-cytochrome C reductase complex subunit UcrQ, putative	-	-1.0428	1.08×10^{-3}
CADAFUAG00002641	Ubiquinol-cytochrome C reductase complex core protein 2, putative	-	-1.0475	5.54×10^{-7}
CADAFUAG00002769	Cytochrome c oxidase subunit Va, putative	-	-1.2279	1.92×10^{-6}
CADAFUAG00003349	Cytochrome c subunit Vb, putative	-	-1.0121	2.06×10^{-6}
CADAFUAG00007360	Mitochondrial F1F0-ATP synthase g subunit, putative	-	-1.1888	6.48×10^{-4}
CADAFUAG00005858	Plasma membrane H^+-ATPase Pma1	-	-1.2169	1.27×10^{-6}

Table S7. Differential gene KEGG enrichment analysis of Δ*choC*-4 vs. Δ*choC* -2.

Gene_ID	Gene description	Regulation	log_2FC	pval
**MAPK signaling pathway**				
CADAFUAG00001412	Protein kinase activator Bem1, putative	+	1.0164	1.47×10^{-4}
CADAFUAG00003688	Rho GTPase ModA, putative	+	1.0462	7.83×10^{-5}
CADAFUAG00003326	Serine/threonine-protein kinase ste20	+	1.0271	1.16×10^{-4}
CADAFUAG00006197	A-pheromone receptor PreA	+	3.448	1.36×10^{-31}
CADAFUAG00000545	Cytokinesis protein SepA/Bni1	+	1.27	2.67×10^{-6}
CADAFUAG00005925	Protein kinase c	+	1.2749	1.26×10^{-6}
CADAFUAG00005058	MAP kinase kinase kinase (Bck1), putative	+	1.5918	3.43×10^{-9}
CADAFUAG00008192	Phosphotransmitter protein Ypd1, putative	+	1.2795	1.75×10^{-6}
CADAFUAG00006950	MAP kinase kinase kinase SskB, putative	+	1.1749	2.43×10^{-5}
**Phosphotidylinnositol signaling system**				
CADAFUAG00002355	1-phosphatidylinositol-3-phosphate 5-kinase (Fab1), putative	+	1.0739	5.31×10^{-5}
CADAFUAG00007510	CDP-diacylglycerol-inositol 3-phosphatidyltransferase PIS	+	1.1352	3.23×10^{-5}
CADAFUAG00007745	Calmodulin	-	1.9247	2.91×10^{-4}
CADAFUAG00005925	Protein kinase c	+	1.2749	1.26×10^{-6}
CADAFUAG00008838	Phosphatidate cytidylyltransferase	+	1.1263	2.67×10^{-5}
CADAFUAG00007699	Inositol monophosphatase QutG, putative	+	4.7279	2.10×10^{-45}
Regulation of Autophagy				
CADAFUAG00004709	Autophagy-related protein 17	-	2.3135	8.58×10^{-9}
CADAFUAG00008080	Serine/threonine-protein kinase atg1	+	1.2168	5.27×10^{-6}
CADAFUAG00003594	Autophagy-related protein 13	+	1.3646	3.40×10^{-7}
CADAFUAG00003192	Autophagy protein Apg6, putative	-	1.5262	6.76×10^{-7}
CADAFUAG00003301	Autophagy ubiquitin-activating enzyme ApgG, putative	+	1.8122	2.59×10^{-7}
**Phagosome**				
CADAFUAG00008238	Calnexin homolog	+	1.0635	3.57×10^{-5}
CADAFUAG00006384	Dynein heavy chain	+	1.4604	5.34×10^{-8}
CADAFUAG00002355	1-phosphatidylinositol-3-phosphate 5-kinase (Fab1), putative	+	1.0739	5.31×10^{-5}
CADAFUAG00002303	NADPH oxidase (NoxA), putative	-	1.1924	4.22×10^{-3}

**Ubiquitin mediated proteolysis**				
CADAFUAG00001899	Ubiquitin-conjugating enzyme E2 2	-	1.331	8.54×10^{-7}
CADAFUAG00008488	Ubiquitin conjugating enzyme, putative	+	1.2683	4.10×10^{-6}
CADAFUAG00001830	Ubiquitin conjugating enzyme, putative	-	1.1053	4.00×10^{-5}
CADAFUAG00004217	Ubiquitin conjugating enzyme, putative	-	1.2938	1.48×10^{-5}
CADAFUAG00005700	Ubiquitin conjugating enzyme, putative	+	1.4171	7.19×10^{-7}
CADAFUAG00001717	Ubiquitin-protein ligase Ufd4, putative	+	1.0585	3.76×10^{-5}
CADAFUAG00007922	Ubiquitin-protein ligase (Tom1), putative	+	1.0867	2.58×10^{-5}
CADAFUAG00006596	Peptidyl-prolyl cis-trans isomerase-like 2	+	1.2862	4.72×10^{-6}
CADAFUAG00003920	U-box domain protein, putative	-	2.2668	7.46×10^{-9}
CADAFUAG00006643	Cell cycle control protein (Cwf8), putative	-	1.1797	1.84×10^{-4}
CADAFUAG00007568	Cell division cycle protein Cdc20, putative	-	1.1269	9.97×10^{-5}
CADAFUAG00004095	Probable E3 ubiquitin ligase complex SCF subunit sconB	-	1.2107	4.43×10^{-6}
CADAFUAG00004971	F-box and WD40 domain protein, putative	+	2.8774	7.01×10^{-24}
CADAFUAG00001710	Cell division control protein Cdc4, putative	+	1.3384	7.81×10^{-7}
**Protein processing in ER**				
CADAFUAG00003937	Translocation protein Sec62, putative	+	1.1408	1.51×10^{-5}
CADAFUAG00006888	Protein translocation complex componenet (Npl1), putative	+	1.7955	3.29×10^{-11}
CADAFUAG00007640	Hsp70 family chaperone Lhs1/Orp150, putative	+	1.6548	1.91×10^{-9}
CADAFUAG00003546	Hsp70 chaperone BiP/Kar2, putative	+	1.6506	6.17×10^{-10}
CADAFUAG00003870	UDP-glucose:glycoprotein glucosyltransferase, putative	+	1.2306	6.88×10^{-6}
CADAFUAG00008238	Calnexin homolog	+	1.0635	3.57×10^{-5}
CADAFUAG00007667	Probable mannosyl-oligosaccharide alpha-1,2-mannosidase 1B	+	3.8973	2.55×10^{-40}
CADAFUAG00001600	Class I alpha-mannosidase 1A	+	1.4965	4.43×10^{-8}
CADAFUAG00006351	Protein kinase (Gcn2), putative	+	1.1049	3.71×10^{-5}
CADAFUAG00004660	Protein kinase, putative	-	1.2354	1.07×10^{-4}
CADAFUAG00008890	Molecular chaperone Hsp70	-	1.2267	2.36×10^{-2}
CADAFUAG00004876	DnaJ domain protein (Mas5), putative	+	1.1494	2.01×10^{-5}
CADAFUAG00009111	Protein mitochondrial targeting protein (Mas1), putative	-	1.0586	7.00×10^{-5}
CADAFUAG00002754	Heat shock protein 90	-	2.0686	2.22×10^{-10}
CADAFUAG00006765	Heat shock protein Hsp20/Hsp26, putative	-	4.8184	1.53×10^{-21}
CADAFUAG00003817	Protein png1	+	1.2267	4.62×10^{-6}
CADAFUAG00001830	Ubiquitin conjugating enzyme, putative	-	1.1053	4.00×10^{-5}
CADAFUAG00004773	AMFR protein, putative	-	1.0491	1.31×10^{-3}
CADAFUAG00003920	U-box domain protein, putative	-	2.2668	7.46×10^{-9}
**Peroxisome**				
CADAFUAG00005376	Peroxisomal membrane protein receptor Pex19, putative	+	1.5482	1.23×10^{-8}
CADAFUAG00002780	Protein sym1	-	2.0383	1.02×10^{-7}

CADAFUAG00009013	Peroxisome biosynthesis protein (Peroxin-2), putative	-	1.0174	9.20×10^{-3}
CADAFUAG00002622	Isopenicillin N-CoA epimerase, putative	-	1.7212	2.31×10^{-3}
CADAFUAG00009385	Acyl-CoA dehydrogenasea family protein	-	1.9411	8.72×10^{-12}
CADAFUAG00009476	Fatty-acyl coenzyme A oxidase (Pox1), putative	-	2.0805	1.23×10^{-7}
CADAFUAG00007131	Long-chain-fatty-acid-CoA ligase, putative	-	3.2319	2.23×10^{-7}
CADAFUAG00004670	Fatty acid activator Faa4, putative	+	1.7823	2.78×10^{-11}
CADAFUAG00002808	AMP-binding enzyme, putative	+	1.8433	2.65×10^{-11}
CADAFUAG00007498	Aminotransferase, class V, putative	+	1.0428	5.96×10^{-5}
CADAFUAG00006035	D-amino acid oxidase	-	1.1201	2.38×10^{-5}
CADAFUAG00001188	3-hydroxymethyl-3-methylglutaryl-Coenzyme A lyase	-	2.1209	3.89×10^{-13}
CADAFUAG00000985	FMN-dependent dehydrogenase family protein	+	5.4528	1.39×10^{-5}
CADAFUAG00008234	Xanthine dehydrogenase HxA, putative	+	2.7639	5.91×10^{-12}
CADAFUAG00002317	AhpC/TSA family protein	-	1.9338	5.56×10^{-4}
CADAFUAG00008176	Superoxide dismutase	-	2.0088	6.18×10^{-5}
CADAFUAG00007458	Superoxide dismutase	+	2.2099	1.64×10^{-3}
CADAFUAG00004037	Catalase	-	1.5848	1.05×10^{-8}
CADAFUAG00000630	Catalase A	+	1.6216	1.28×10^{-7}
CADAFUAG00003757	Catalase, putative	+	5.3597	9.31×10^{-28}
**Proteasome**				
CADAFUAG00002328	Proteasome regulatory particle subunit (RpnI), putative	-	1.169	1.86×10^{-5}
CADAFUAG00009176	Proteasome regulatory particle subunit (RpnF), putative	-	1.4819	2.98×10^{-3}
CADAFUAG00001681	Proteasome regulatory particle subunit (RpnG), putative	-	1.465	1.34×10^{-7}
CADAFUAG00005131	Proteasome regulatory particle subunit Rpt1, putative	-	1.0909	4.49×10^{-5}
CADAFUAG00008968	Proteasome regulatory particle subunit Rpt5, putative	-	1.0164	1.2×10^{-4}
CADAFUAG00005585	Proteasome component Prs2, putative	-	1.5431	1.18×10^{-2}
CADAFUAG00009664	Proteasome subunit beta type	-	1.365	4.90×10^{-3}
CADAFUAG00009519	Proteasome subunit alpha type	-	1.6357	1.22×10^{-6}
CADAFUAG00002607	Proteasome component Pre6, putative	-	1.1189	3.01×10^{-5}
CADAFUAG00008315	Proteasome subunit beta type	-	1.2027	7.21×10^{-3}
CADAFUAG00004270	Proteasome component Pup2, putative	-	1.8354	7.68×10^{-3}
**Meiosis yeast**				
CADAFUAG00004157	MFS monosaccharide transporter, putative	-	8.3246	1.89×10^{-35}
CADAFUAG00002192	Adenylate cyclase AcyA	+	1.0594	5.97×10^{-5}
CADAFUAG00004343	Serine threonine protein kinase, putative	+	1.0453	6.48×10^{-5}
CADAFUAG00004724	APSES transcription factor StuA	+	2.1855	7.16×10^{-16}
CADAFUAG00003650	Serine/threonine-protein phosphatase	+	1.6934	2.63×10^{-10}
CADAFUAG00008947	Serine/threonine-protein phosphatase	+	1.2392	3.20×10^{-6}

CADAFUAG00005672	Structural maintenance of chromosomes protein	+	1.1721	1.29×10^{-5}
CADAFUAG00002666	DNA damage repair protein (Rad9), putative	+	1.0551	2.50×10^{-4}
CADAFUAG00007568	Cell division cycle protein Cdc20, putative	-	1.1269	9.97×10^{-5}
CADAFUAG00002132	Checkpoint protein kinase (SldA), putative	-	1.1512	4.44×10^{-5}
CADAFUAG00002777	Meiosis-specific topoisomerase Spo11, putative	+	1.4497	1.99×10^{-6}
CADAFUAG00002350	Putative uncharacterized protein	+	1.0242	6.41×10^{-4}
CADAFUAG00005114	DNA replication licensing factor Mcm2, putative	+	1.9131	3.21×10^{-12}
CADAFUAG00002825	DNA replication licensing factor Mcm5, putative	+	1.9682	2.02×10^{-10}
CADAFUAG00004775	DNA replication licensing factor Mcm4, putative	+	1.8276	1.58×10^{-11}
CADAFUAG00009054	DNA replication licensing factor Mcm3, putative	+	1.8756	1.29×10^{-11}
**Cell cycle**				
CADAFUAG00007568	Cell division cycle protein Cdc20, putative	-	1.1269	9.97×10^{-5}
CADAFUAG00001710	Cell division control protein Cdc4, putative	+	1.3384	7.81×10^{-7}
CADAFUAG00004095	Probable E3 ubiquitin ligase complex SCF subunit sconB	-	1.2107	4.43×10^{-6}
CADAFUAG00005067	Checkpoint protein kinase, putative	-	1.7463	5.07×10^{-10}
CADAFUAG00001802	DASH complex component Dam1, putative	-	1.5161	7.62×10^{-5}
CADAFUAG00002132	Checkpoint protein kinase (SldA), putative	-	1.1512	4.44×10^{-5}
CADAFUAG00001771	Nuclear pore complex subunit, putative	-	1.0901	7.31×10^{-5}
CADAFUAG00002666	DNA damage repair protein (Rad9), putative	+	1.0551	2.50×10^{-4}
CADAFUAG00005672	Structural maintenance of chromosomes protein	+	1.1721	1.29×10^{-5}
CADAFUAG00004730	Structural maintenance of chromosomes protein	+	1.0296	7.64×10^{-5}
Glycerophospholipid metabolism				
CADAFUAG00003901	1-acylglycerol-3-phosphate acyltransferase (AtaAp), putative	+	1.1256	4.81×10^{-5}
CADAFUAG00004565	Phospholipase D (PLD), putative	-	1.0755	5.98×10^{-5}
CADAFUAG00005036	Phospholipase D1 (PLD1), putative	+	1.0792	4.67×10^{-5}
CADAFUAG00005063	Phosphatidate cytidylyltransferase, putative	+	1.1648	1.53×10^{-5}
CADAFUAG00007261	Phospholipid methyltransferase	-	2.4652	2.02×10^{-17}
CADAFUAG00000092	Phosphatidylglycerol specific phospholipase, putative	+	3.2422	6.97×10^{-30}
CADAFUAG00002626	Lysophospholipase 2	+	2.7865	3.36×10^{-4}
CADAFUAG00005659	Lysophospholipase 3	-	1.3451	5.96×10^{-7}
CADAFUAG00007260	Ethanolamine kinase, putative	-	2.2114	4.29×10^{-15}
CADAFUAG00008541	Phosphoethanolamine	-	1.8392	7.20×10^{-11}
CADAFUAG00005887	Aminoalcoholphosphotransferase	-	1.1047	1.43×10^{-4}
CADAFUAG00007631	Phosphatidylserine decarboxylase, putative	-	2.2005	6.12×10^{-8}
CADAFUAG00008838	Phosphatidate cytidylyltransferase	+	1.1263	2.67×10^{-5}
CADAFUAG00007510	CDP-diacylglycerol-inositol 3-phosphatidyltransferase PIS	+	1.1352	3.23×10^{-5}
CADAFUAG00003739	Glycerophosphoryl diester phosphodiesterase family protein	-	3.1092	2.31×10^{-5}
CADAFUAG00004684	Tafazzin	-	1.1138	8.89×10^{-5}
CADAFUAG00008918	Alpha/beta hydrolase, putative	-	1.0393	1.23×10^{-4}

**Glycosphingolipid biosynthesis**				
CADAFUAG00002838	Probable alpha-galactosidase B	-	1.9638	1.78×10^{-11}
CADAFUAG00006352	Probable alpha-galactosidase A	-	1.1921	8.96×10^{-4}
CADAFUAG00003087	Beta-hexosaminidase	+	3.0671	7.83×10^{-27}
CADAFUAG00003685	Beta-N-hexosaminidase, putative	+	5.2034	3.82×10^{-50}
**Sphingolipid metabolism**				
CADAFUAG00008112	Longevity-assurance protein (LAC1), putative	+	1.0835	1.55×10^{-4}
CADAFUAG00008744	Neutral/alkaline nonlysosomal ceramidase, putative	+	2.1255	4.06×10^{-14}
CADAFUAG00007016	Sphinganine hydroxylase Sur2, putative	-	1.1916	1.39×10^{-3}
CADAFUAG00008112	Longevity-assurance protein (LAC1), putative	+	1.0835	1.55×10^{-4}
CADAFUAG00000139	Probable beta-galactosidase E	-	1.0226	5.91×10^{-3}
CADAFUAG00007691	Probable beta-galactosidase A	-	1.0626	1.69×10^{-3}
CADAFUAG00008210	Sphingolipid desaturase, putative	-	1.5121	3.89×10^{-6}
CADAFUAG00006372	Ceramide glucosyltransferase, putative	-	1.1346	4.58×10^{-4}
CADAFUAG00003262	Beta-1,6-glucanase Neg1, putative	+	2.2854	1.62×10^{-6}
CADAFUAG00008433	Extracellular sialidase/neuraminidase, putative	-	1.8526	3.30×10^{-4}
CADAFUAG00007809	Acid sphingomyelinase, putative	+	1.1247	6.26×10^{-5}
CADAFUAG00002838	Probable alpha-galactosidase B	-	1.9638	1.78×10^{-11}
CADAFUAG00006352	Probable alpha-galactosidase A	-	1.1921	8.96×10^{-4}
CADAFUAG00006736	Arylsulfatase, putative	+	2.8611	2.54×10^{-24}
CADAFUAG00000945	Arylsulfatase, putative	-	1.03	5.09×10^{-3}
**Oxidative phosphorylation**				
CADAFUAG00009113	Putative uncharacterized protein	-	1.6112	3.14×10^{-6}
CADAFUAG00004559	NADH-ubiquinone dehydrogenase 24 kDa subunit, putative	-	1.2025	8.90×10^{-6}
CADAFUAG00003449	NADH-ubiquinone oxidoreductase 18 kDa subunit, putative	-	1.4734	5.05×10^{-8}
CADAFUAG00002749	NADH-ubiquinone oxidoreductase	-	1.303	1.46×10^{-6}
CADAFUAG00001231	Putative uncharacterized protein	-	1.1309	2.67×10^{-5}
CADAFUAG00007123	NADH-ubiquinone oxidoreductase 19 kDa subunit, putative	-	1.4404	1.19×10^{-7}
CADAFUAG00007790	Ubiquinol-cytochrome c reductase complex 17 kd protein	-	1.7151	2.29×10^{-7}
CADAFUAG00008495	Cytochrome b-c1 complex subunit 7	-	1.601	7.95×10^{-7}
CADAFUAG00007399	Ubiquinol-cytochrome C reductase complex subunit UcrQ, putative	-	1.6183	4.70×10^{-5}
CADAFUAG00006375	Cytochrome c oxidase subunit V	-	2.1917	1.03×10^{-15}
CADAFUAG00005824	Cytochrome c oxidase subunit VIa, putative	-	2.3615	7.43×10^{-18}
CADAFUAG00005595	Cytochrome c oxidase subunit 7A	-	2.0095	1.73×10^{-13}
CADAFUAG00002769	Cytochrome c oxidase subunit Va, putative	-	1.8104	2.82×10^{-11}
CADAFUAG00003349	Cytochrome c subunit Vb, putative	-	2.2451	3.66×10^{-16}
CADAFUAG00008447	Cytochrome c subunit, putative	-	1.8381	7.36×10^{-12}
CADAFUAG00004736	Cytochrome c oxidase polypeptide vib	-	1.7659	5.17×10^{-11}

CADAFUAG00001607	Inorganic diphosphatase, putative	+	2.4928	$4.10×10^{-9}$
CADAFUAG00007987	ATP synthase subunit ATP9, putative	-	2.2271	$5.29×10^{-16}$
CADAFUAG00000687	ATP synthase D chain, mitochondrial, putative	-	1.4123	$1.07×10^{-2}$
CADAFUAG00003742	Mitochondrial F1F0 ATP synthase subunit F (Atp17), putative	-	1.2784	$1.37×10^{-6}$
CADAFUAG00004553	Mitochondrial ATP synthase epsilon chain domain-containing protein	-	1.0512	$2.82×10^{-3}$

Table S8. Differential gene KEGG enrichment analysis of WT-4 vs. WT-2.

Gene ID	Description	Regulation	log_2FC	p-value
**MAPK signaling pathway**				
CADAFUAG00003688	Rho GTPase ModA, putative	+	0.56063	$7.6649×10^{-3}$
CADAFUAG00000545	Cytokinesis protein SepA/Bni1	+	0.80927	$1.4501×10^{-4}$
CADAFUAG00005058	MAP kinase kinase kinase (Bck1), putative	+	0.69132	$1.1265×10^{-3}$
CADAFUAG00008287	MAP kinase MpkA	+	0.60985	$3.1051×10^{-3}$
CADAFUAG00002297	Filament-forming protein (Tpr/p270), putative	+	0.79499	$1.7281×10^{-4}$
CADAFUAG00006950	MAP kinase kinase kinase SskB, putative	+	0.61739	$3.3869×10^{-3}$
CADAFUAG00003688	Rho GTPase ModA, putative	+	0.56063	$7.6649×10^{-3}$
CADAFUAG00002199	Lactoylglutathione lyase	+	0.54226	$1.1417×10^{-2}$
CADAFUAG00006525	Glyoxalase family protein	-	-0.69577	$5.2671×10^{-3}$
CADAFUAG00007322	MAP kinase kinase (Pbs2), putative	-	-0.77727	$3.0374×10^{-4}$
CADAFUAG00006975	Mitogen-activated protein kinase hog1	-	-0.94718	$8.45×10^{-6}$
CADAFUAG00008714	Transcription factor AbaA	-	-1.8441	$6.02×10^{-6}$
**Phosphatidylinositol signaling system**				
CADAFUAG00009531	APSES transcription factor (MbpA), putative	+	0.81025	$2.2757×10^{-4}$
CADAFUAG00007273	1-phosphatidylinositol-4,5-bisphosphate phosphodiesterase Plc1, putative	+	1.8554	$3.06×10^{-10}$
**Regulation of Autophagy**				
CADAFUAG00005966	Vacuolar protein 8	+	0.56054	$7.4197×10^{-3}$
CADAFUAG00003301	Autophagy ubiquitin-activating enzyme ApgG, putative	+	1.0924	$2.18×10^{-7}$
CADAFUAG00009234	Autophagy-related protein 8	+	0.55954	$7.4132×10^{-3}$
**Phagosome**				
CADAFUAG00000607	Actin Act1	+	0.60213	$3.2342×10^{-3}$
CADAFUAG00008065	RAB GTPase Ypt5, putative	+	0.71431	$6.2857×10^{-4}$

CADAFUAG00008238	Calnexin homolog		0.72154	5.5755×10^{-4}
CADAFUAG00003796	Vacuolar protein sorting-associated protein 27	+	0.61287	3.7653×10^{-3}
CADAFUAG00006543	Rab small monomeric GTPase Rab7, putative	+	0.7428	5.6552×10^{-4}
CADAFUAG00007900	SNARE domain protein	+	0.56318	9.2801×10^{-3}
CADAFUAG00006384	Dynein heavy chain	+	0.83673	9.68×10^{-5}
CADAFUAG00003913	Tubulin subunit TubB	+	0.55833	9.9429×10^{-3}
CADAFUAG00009262	Vacuolar sorting receptor (Mrl1), putative	+	0.83653	7.90×10^{-5}
CADAFUAG00005223	Protein translocation complex subunit Sss1, putative	+	0.62236	7.9304×10^{-3}
CADAFUAG00005456	Rho GTPase Rac, putative	+	0.54159	1.0598×10^{-2}
**Ubiquitin mediated proteolysis**				
CADAFUAG00003933	Poly(A)+ RNA transport protein (UbaA), putative	+	0.54763	7.4338×10^{-3}
CADAFUAG00007851	SUMO conjugating enzyme (UbcI), putative	+	0.68262	1.8608×10^{-3}
CADAFUAG00005443	Ubiquitin conjugating enzyme (UbcB), putative	+	1.2085	1.39×10^{-7}
CADAFUAG00004384	Ubiquitin conjugating enzyme, putative	+	1.2036	4.1692×10^{-3}
CADAFUAG00006447	Ubiquitin conjugating enzyme (UbcA), putative	+	1.4108	1.19×10^{-10}
CADAFUAG00001717	Ubiquitin-protein ligase Ufd4, putative	+	0.6888	1.0032×10^{-3}
CADAFUAG00007922	Ubiquitin-protein ligase (Tom1), putative	+	0.51785	1.2795×10^{-2}
CADAFUAG00003920	U-box domain protein, putative	+	0.97866	1.29×10^{-5}
CADAFUAG00006107	E3 ubiquitin ligase complex SCF subunit sconC	+	0.67294	1.4096×10^{-3}
CADAFUAG00002240	SCF ubiquitin ligase subunit CulC, putative	+	1.1352	8.94×10^{-6}
CADAFUAG00007355	Anaphase promoting complex subunit 10 (APC10), putative	+	0.75584	1.9342×10^{-3}
CADAFUAG00008051	F-box and WD domain protein	+	0.50361	1.5229×10^{-2}
CADAFUAG00006643	Cell cycle control protein (Cwf8), putative	-	-0.51392	1.4696×10^{-2}
CADAFUAG00001195	Anaphase promoting complex subunit Apc11, putative	-	-0.66252	3.87×10^{-3}
CADAFUAG00004095	Probable E3 ubiquitin ligase complex SCF subunit sconB	-	-1.4857	5.14×10^{-12}
**Protein processing in ER**				
CADAFUAG00005223	Protein translocation complex subunit Sss1, putative	+	0.62236	7.9304×10^{-3}

CADAFUAG00008238	Calnexin homolog	+	0.72154	5.5755×10^{-4}
CADAFUAG00002054	Mannosyl-oligosaccharide alpha-1,2-mannosidase	+	0.54053	1.5976×10^{-2}
CADAFUAG00004123	Lectin family integral membrane protein, putative	+	1.2108	7.4494×10^{-4}
CADAFUAG00003546	Hsp70 chaperone BiP/Kar2, putative	+	0.7571	3.1329×10^{-4}
CADAFUAG00004529	DnaJ domain protein, putative	+	0.71583	1.2142×10^{-3}
CADAFUAG00004122	Cell division control protein Cdc48	+	1.0083	1.42×10^{-6}
CADAFUAG00005903	UV excision repair protein (RadW), putative	+	1.0197	9.94×10^{-7}
CADAFUAG00009111	Protein mitochondrial targeting protein (Mas1), putative	+	1.0957	2.55×10^{-7}
CADAFUAG00005443	Ubiquitin conjugating enzyme (UbcB), putative	+	1.2085	1.39×10^{-7}
CADAFUAG00004660	Protein kinase, putative	-	-1.134	2.23×10^{-5}
**Snare interactions in vesicular transport**				
CADAFUAG00000456	SNARE protein Snc2, putative	+	0.74919	4.4014×10^{-4}
CADAFUAG00005547	ER-Golgi SNARE complex subunit (Sed5), putative	+	0.98847	4.10×10^{-6}
CADAFUAG00007900	SNARE domain protein	+	0.56318	9.2801×10^{-3}
**Peroxisome**				
CADAFUAG00006972	Peroxisome biosynthesis protein (Peroxine-7), putative	-	-1.1288	5.34×10^{-7}
CADAFUAG00004915	Peroxisome biosynthesis protein (PAS1/Peroxin-1), putative	-	-0.52547	1.5472×10^{-2}
CADAFUAG00006550	Peroxisomal membrane protein (Pex3), putative	-	-1.1895	6.26×10^{-8}
CADAFUAG00009013	Peroxisome biosynthesis protein (Peroxin-2), putative	-	-0.88768	1.7883×10^{-3}
CADAFUAG00009341	Peroxisome biosynthesis protein (Peroxin-10), putative	-	-1.3336	1.22×10^{-5}
CADAFUAG00002705	Peroxisomal membrane protein Pmp47, putative	-	-1.3739	1.20×10^{-10}
CADAFUAG00007053	ABC fatty acid transporter, putative	-	-0.8572	1.1094×10^{-4}
CADAFUAG00007131	Long chain fatty acid CoA ligase, putative	-	-3.4394	8.10×10^{-21}
CADAFUAG00006035	D-amino acid oxidase	-	-1.7319	4.0627×10^{-4}
CADAFUAG00005587	Enoyl-CoA hydratase/isomerase family protein	+	0.58613	9.5172×10^{-3}
CADAFUAG00007892	Mevalonate kinase	+	1.4028	1.0057×10^{-3}
**Proteasome**				

CADAFUAG00008070	26S proteasome regulatory subunit Rpn2, putative	+	0.75416	3.8946×10^{-4}
CADAFUAG00004983	Proteasome regulatory particle subunit (RpnC), putative	+	0.71833	5.9666×10^{-4}
CADAFUAG00005649	Proteasome regulatory particle subunit (RpnE), putative	+	0.62921	3.2665×10^{-3}
CADAFUAG00003854	Proteasome regulatory particle subunit (RpnK), putative	+	0.65653	2.2866×10^{-3}
CADAFUAG00005629	Proteasome regulatory particle subunit (RpnL), putative	+	0.7974	3.7282×10^{-3}
CADAFUAG00007228	26S proteasome complex subunit Sem1, putative	+	0.6975	1.1163×10^{-3}
CADAFUAG00001856	Proteasome regulatory particle subunit Rpt4, putative	+	0.97539	6.87×10^{-6}
CADAFUAG00000612	Proteasome subunit alpha type	+	1.0331	2.2158×10^{-4}
**Meiosis**				
CADAFUAG00002192	Adenylate cyclase AcyA	+	0.54405	9.3249×10^{-3}
CADAFUAG00004390	NDT80 / PhoG like DNA-binding family protein	+	0.63734	2.0151×10^{-3}
CADAFUAG00005672	Structural maintenance of chromosomes protein	+	0.8179	1.1297×10^{-4}
CADAFUAG00007355	Anaphase promoting complex subunit 10 (APC10),	+	0.75584	1.9342×10^{-3}
CADAFUAG00009285	Origin recognition complex subunit Orc5, putative		0.77429	5.4805×10^{-4}
CADAFUAG00002350	Putative uncharacterized protein		0.62044	9.3924×10^{-3}
CADAFUAG00004297	Protein kinase, putative	-	-1.0772	3.42×10^{-5}
CADAFUAG00001195	Anaphase promoting complex subunit Apc11, putative	-	-0.66252	3.87×10^{-3}
CADAFUAG00002777	Meiosis-specific topoisomerase Spo11, putative	-	-0.88286	4.2873×10^{-3}
CADAFUAG00006531	Origin recognition complex subunit 2, putative	-	-1.5214	5.70×10^{-12}
**Cell cycle**				
CADAFUAG00006107	E3 ubiquitin ligase complex SCF subunit sconC	+	0.67294	1.4096×10^{-3}
CADAFUAG00000696	Transcriptional repressor TupA/RocA, putative	+	1.2522	2.59×10^{-9}
CADAFUAG00001706	GTP binding protein (SPG1), putative	+	0.87426	6.64×10^{-5}
CADAFUAG00005672	Structural maintenance of chromosomes protein	+	0.8179	1.1297×10^{-4}
CADAFUAG00009285	Origin recognition complex subunit Orc5,	+	0.77429	5.4805×10^{-4}

	putative			
CADAFUAG00002350	Putative uncharacterized protein	+	0.62044	9.3924×10^{-3}
CADAFUAG00002178	G1/S regulator NimO, putative	-	-0.65299	4.1587×10^{-3}
CADAFUAG00005067	Checkpoint protein kinase, putative	-	-0.99523	2.31×10^{-5}
CADAFUAG00001802	DASH complex component Dam1, putative	-	-0.75611	2.602×10^{-3}
CADAFUAG00008384	G2/M-specific cyclin NimE	-	-0.59472	4.5434×10^{-3}
CADAFUAG00002027	Cell cycle control protein tyrosine phosphatase Mih1, putative	-	-0.5922	1.1366×10^{-2}
CADAFUAG00004095	Probable E3 ubiquitin ligase complex SCF subunit sconB	-	-1.4857	5.14×10^{-12}
CADAFUAG00004297	Protein kinase, putative	-	-1.0772	3.42×10^{-5}
CADAFUAG00006202	DNA damage and replication checkpoint protein Rfx1, putative	-	-0.62117	6.8163×10^{-3}
CADAFUAG00008265	Cyclin dependent kinase inhibitor Pho81, putative	-	-0.58424	7.7603×10^{-3}
CADAFUAG00006531	Origin recognition complex subunit 2, putative	-	-1.5214	5.70×10^{-12}
**Glycerophospholipid metabolism**				
CADAFUAG00002626	Lysophospholipase 2	+	4.7086	1.48×10^{-12}
CADAFUAG00000092	Phosphatidylglycerol specific phospholipase, putative	+	1.2352	4.47×10^{-9}
CADAFUAG00008884	Choline kinase, putative	+	0.9131	1.2408×10^{-2}
CADAFUAG00005063	Phosphatidate cytidylyltransferase, putative	+	0.55927	9.677×10^{-3}
CADAFUAG00003739	Glycerophosphoryl diester phosphodiesterase family protein	+	0.86469	7.1393×10^{-4}
CADAFUAG00008918	Alpha/beta hydrolase, putative	+	0.75329	8.732×10^{-3}
CADAFUAG00004565	Phospholipase D (PLD), putative	+	1.1864	9.18×10^{-5}
CADAFUAG00005036	Phospholipase D1 (PLD1), putative	+	0.50469	1.6981×10^{-2}
CADAFUAG00007184	Phosphatidylserine decarboxylase, putative	+	0.6251	3.0997×10^{-3}
CADAFUAG00004416	Glycerol 3-phosphate dehydrogenase (GfdB), putative	-	-1.1005	9.29×10^{-5}
CADAFUAG00007839	Glycerol-3-phosphate acyltransferase Sct1, putative	-	-1.1374	8.99×10^{-8}
CADAFUAG00007261	Phospholipid methyltransferase	-	-0.91194	8.98×10^{-5}
CADAFUAG00004056	Phosphatidylethanolamine N-methyltransferase	-	-0.54358	9.7073×10^{-3}
CADAFUAG00004684	Tafazzin	-	-1.1476	4.58×10^{-6}

CADAFUAG00009512	Phospholipase PldA, putative	-	-2.5557	8.00×10^{-31}
CADAFUAG00001053	Phosphatidylserine decarboxylase family protein	-	-1.4839	6.3127×10^{-3}
**Glycerolipid metabolism**				
CADAFUAG00006222	Dihydroxyacetone kinase (DakA), putative	+	0.94338	8.17×10^{-6}
CADAFUAG00001170	Aldehyde dehydrogenase, putative	+	0.57471	5.7277×10^{-3}
CADAFUAG00001367	Putative uncharacterized protein	-	-1.2679	1.44×10^{-8}
CADAFUAG00006869	Aldehyde reductase (AKR1), putative	-	-0.50065	1.543×10^{-2}
CADAFUAG00007839	Glycerol-3-phosphate acyltransferase Sct1, putative	-	-1.1374	8.99×10^{-8}
CADAFUAG00006243	Lipase, putative	-	-1.3606	4.96×10^{-5}
CADAFUAG00003816	Alkaline lipase, putative	-	-1.9156	1.45×10^{-7}
CADAFUAG00005204	Phospholipid:diacylglycerol acyltransferase, putative	-	-0.56329	1.4016×10^{-2}
CADAFUAG00006352	Probable alpha-galactosidase A	-	-0.85125	7.247×10^{-4}
